# PRODUCES+: Guidance for co-creation in public health informed by evidence and user experience

**DOI:** 10.1016/j.puhip.2026.100825

**Published:** 2026-07-08

**Authors:** Danielle Marie Agnello, Giuliana Raffaella Longworth, Maria Giné-Garriga, Sebastien Chastin, Dawn A. Skelton, Calum F. Leask

**Affiliations:** a School of Health and Life Sciences, Glasgow Caledonian University, Glasgow, United Kingdom; b Global Health Section, Department of Public Health, University of Copenhagen, Denmark; c Department of Physical Activity and Sport Sciences, Faculty of Psychology, Education and Sport Sciences Blanquerna, Universitat Ramon Llull, Barcelona, Spain; d Department of Physical Therapy, Faculty of Health Sciences Blanquerna, Universitat Ramon Llull, Barcelona, Spain; e Department of Movement and Sports Sciences, Ghent University, Ghent, Belgium; f Aberdeen City Health and Social Care Partnership, NHS Grampian, Aberdeen, United Kingdom

**Keywords:** Co-creation, Co-design, Co-production, Participatory, Guidance, Framework, Implementation, Evaluation, Methodology, Method, Public health

## Abstract

**Objectives:**

Despite the growing popularity of co-creation to develop complex interventions, there is limited guidance on planning and implementing a co-creation process in public health. This study aims to critically review and update one such guidance, the PRODUCES framework, to develop a more comprehensive, user-friendly guide for public health researchers and practitioners.

**Study design:**

Evidence- and user-informed guideline development.

**Methods:**

A multi-method, evidence-informed approach was used to identify strengths, gaps, and areas for improvement. This included a snowballing review of peer-reviewed articles citing the PRODUCES framework, followed by in-depth interviews and a mixed-methods survey with lead authors identified through the review. Data from included studies, interviews, and survey responses were analysed thematically.

**Results:**

The search identified 96 studies, of which 21 were included. Four interviews and nine survey responses were obtained. Strengths included flexibility, clear foundational guidance, and practical applicability, while limitations related to clarity, overlapping stages, and terminology. Participants suggested strengthening implementation guidance and methods and highlighted the value of complementary frameworks. These findings informed the development of the PRODUCES+ guideline.

**Conclusions:**

Building on user input, the original framework, and contemporary co-creation literature, the PRODUCES+ Guideline provides an expanded, step-by-step resource for planning co-creation in public health.

## 1 Introduction

Co-creation is any act of collective creativity that involves a broad range of relevant and affected actors in creative problem-solving that aims to produce a desired outcome [1]. The use of co-creation in public health has grown and is becoming an increasingly prevalent methodology, with an exponential growth in publications mentioning co-creation since 2010 [1,2]. Co-creation is progressively demanded by funders, governments, and policymakers to democratise and accelerate research impact [1], enhance health interventions and tackle wicked problems. Consequently, establishing a robust evidence base is crucial to ensuring that co-creation is both trustworthy and effective for public health [3].

However, a clear, unified, step-by-step approach to co-creating interventions is lacking [1,4,5]. Before Leask et al. developed the PRODUCES framework, no precise or systematic framework existed to plan, develop, or evaluate co-created public health interventions [5]. Rather than developing a new framework, this study sought to refine and extend an existing evidence-informed approach. PRODUCES was selected as the basis for this work because it is the only known framework specifically developed to guide the planning and evaluation of co-created public health interventions and has been widely cited and applied since its publication. Building on an established framework allows the retention of its strengths while addressing identified challenges, thereby supporting the development of more accessible guidance without introducing an additional standalone framework.

Several authors of this study had experience applying the PRODUCES framework in their research, providing insight into its practical use and areas requiring further development. This study therefore sets out to critically review and refine the framework to enhance its clarity, usability, and value across multiple contexts. This work is also part of the Health CASCADE network, a multidisciplinary consortium of researchers working to establish co-creation as a scientific methodology for public health by drawing on evidence and approaches from all disciplines that apply co-creation.

### 1.1 Aims and scope

**Fig. 1:**
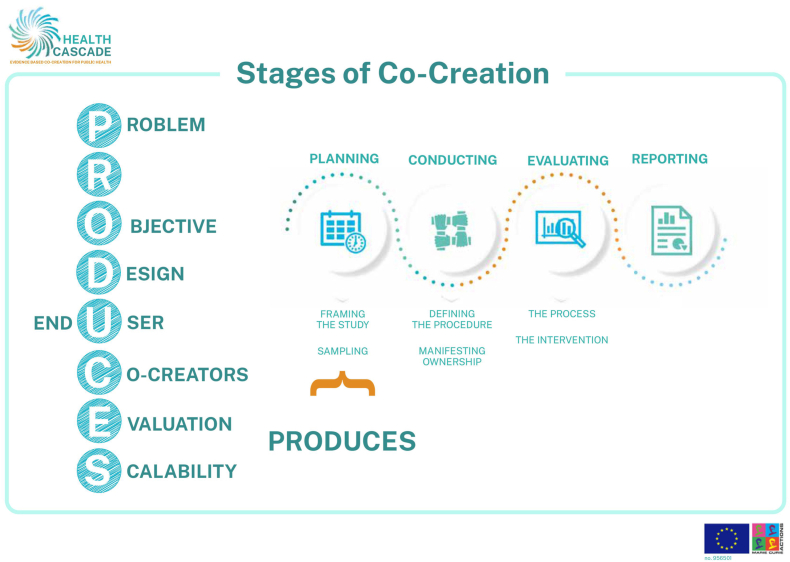
Infographic visualising the stages and principles of co-creation, and details from the PRODUCES framework (source = Health CASCADE, 2022) [6].

This study aims to critically review the PRODUCES framework [5] to identify gaps, strengths, and areas for improvement and generate user-friendly guidance for co-creation. This updated guidance can assist in planning, conducting, evaluating, and reporting co-creation processes, providing step-by-step, practical instructions. Fig. 1 visualizes the four stages and six principles of the PRODUCES framework.

This study aims to answer the following research questions:

1. What are the key strengths of the PRODUCES framework?
2. What are the gaps in the PRODUCES framework?
3. What is further needed for a revised guideline for planning co-creation for public health interventions?

## 2 Methods

### 2.1 Study design

This study aimed to develop a Co-Creation Guideline for Public Health based on user feedback and supported by co-creation literature.

Gaps, strengths, and areas for improvement were identified using a multi-method approach: a) a snowballing review of publications citing Leask et al., 2019; b) semi-structured interviews with included authors who referenced the principles or stages of the PRODUCES framework; and c) a mixed-methods survey distributed to included authors who cited the framework.

### 2.2 Snowballing review

A snowballing review is a method that identifies relevant studies by tracking citations forward (citation tracking) or backwards (reference screening) to find additional literature connected to a key publication [7]. In this study, a forward citation tracking approach was applied. We identified all articles citing Leask et al. [5] between 1 January 2019 and 1 January 2023 using PubMed Central, which allows citation searching within the PubMed database. The list of citing articles was exported as a Comma-Separated Values file from PubMed and then screened for relevance using the selection criteria in Table 1. The two lead researchers (DMA and GRL) independently conducted the screening, resolving any conflicts through discussion.

**Table 1:** The inclusion criteria for full-text screening.

Inclusion Criteria
The paper reported applying, using, or following the PRODUCES framework in the study
The paper was written in English

Data was extracted by two researchers(DMA and GRL) using a pre-defined Google Forms template. The extraction form captured details such as the paper's title, mentions of the PRODUCES framework's stages, principles, or definition of co-creation, and any reported strengths and weaknesses.

### 2.3 Interviews and surveys

Among the included papers, lead authors were invited to an interview if their publication explicitly discussed the application of one or more stages or principles of the PRODUCES framework, allowing for more in-depth exploration of their practical experiences. Potential interviewees were invited via email, and those who agreed to participate provided informed consent, following the ethical approval granted for this study. Authors who were not selected for, or did not participate in, an interview were invited to complete a survey to capture perspectives from the remaining included publications and broaden the range of experiences represented.

The interview was conducted online using Microsoft Teams Meeting software [8] and was guided by a pre-defined interview guide. This guide was designed using the criteria derived from Lobczowska et al.’s criteria for selection: “practicality,” defined as understandability and clarity of key constructs, ease of use, and comprehensiveness in terms of coverage of adaptation and evaluation recommendations [9]. The interview guide was piloted by interviewing a co-creation researcher (CFL) and then revised accordingly.

The survey was derived directly from the interview guide, with quantitative and qualitative questions adapted into a structured format to facilitate broader data collection. The survey was sent to the respondents using Google Forms [10]. The interview guide and survey questions can be found in Supplementary Files 1 and 2, respectively.

### 2.4 Analysis

#### 2.4.1 Quantitative data

Satisfaction scores ranging from 1 (strongly dissatisfied) to 5 (very satisfied), and the overall rating of the PRODUCES framework were aggregated from the interviews and survey responses and depicted in tables.

#### 2.4.2 Qualitative data

An inductive thematic analysis of both the interview and survey response data was undertaken following the six stages outlined by Braun and Clarke [11]. This involved reading and re-reading transcripts and papers to ensure familiarisation with the data, generating initial codes, organising identified codes, and developing overarching themes before reviewing and finalising themes [12].

Two researchers (DMA and GRL) independently coded the interview transcripts and survey responses inductively and then met to develop a preliminary thematic framework. This was undertaken by extrapolating identified codes to a higher level of abstraction by examining similarities and differences between codes and considering relationships between codes. The initial framework was discussed with senior researchers (MGG, SC, DAS, and CFL) to identify any discrepancies or challenges. Themes were refined through discussion among the research team, with codes merged, redefined, or reorganised where necessary to improve conceptual clarity and ensure alignment with the underlying data.

Once agreed, the subsequent framework was applied to the remaining transcripts and survey responses by two researchers (DMA and GRL). Further iterative modifications were made to the framework during this process, which included the removal of duplicate codes, re-categorisation, and the addition of new codes as new data were analysed.

Given that some members of the research team were involved in the original development of the PRODUCES framework, reflexivity was considered during interpretation. Also, the researchers who led the analysis (DMA and GRL) were not involved in the PRODUCES framework's development, while senior researchers (MGG, SC, DAS, and CFL) with prior involvement contributed to theme discussions through critical reflection on their perspectives and potential influence.

Finally, as the qualitative component aimed to inform guideline refinement through targeted insights from PRODUCES users, thematic saturation was not used as a criterion for sample adequacy. Instead, findings were triangulated across the literature review, interviews, and survey responses.

#### 2.4.3 Data synthesis

Findings from the snowballing review, interviews, and survey responses were synthesised and triangulated to identify strengths, limitations, and opportunities for improvement in the PRODUCES framework. These were mapped to inform areas for preservation, clarification, and revision in the PRODUCES+ Guideline.

## 3 Results

### 3.1 Snowballing search

**Fig. 2:**
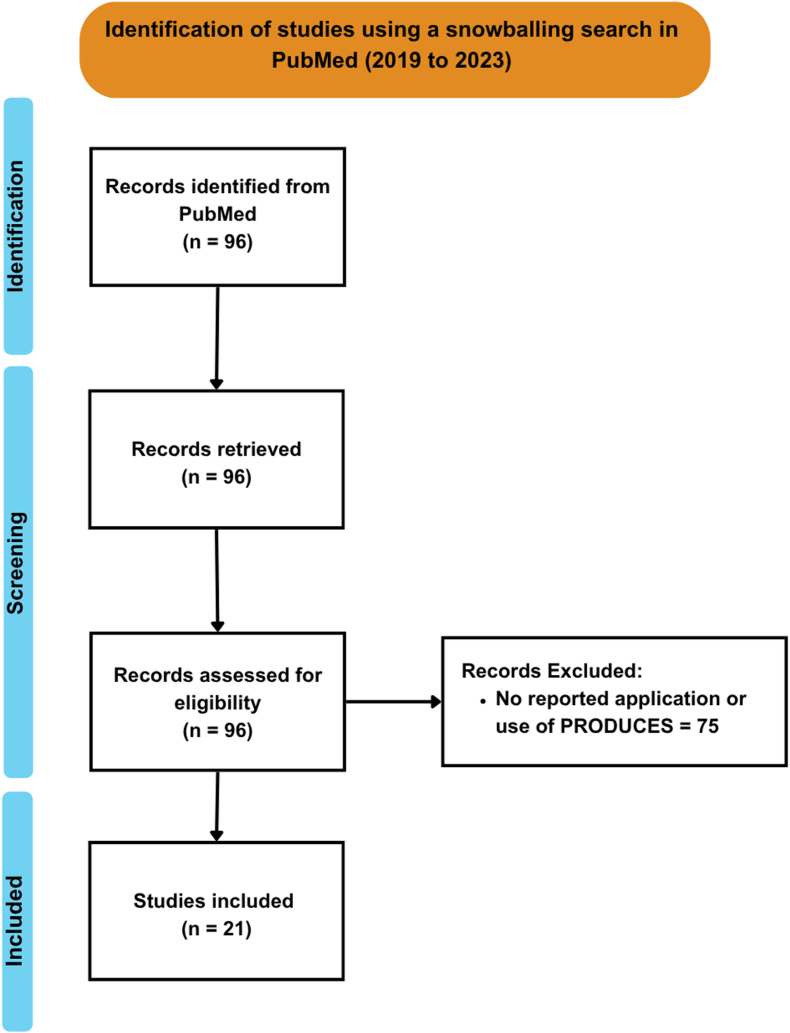
PRISMA-like flow chart of Snowball search results.

From the snowballing search performed, 96 studies were identified, and after screening for relevance, 21 studies were taken to the extraction step. Fig. 2 visualizes the screening process in a PRISMA-like flow chart.

The 21 included studies were reviewed, and data were extracted to understand how the PRODUCES framework had been applied in practice, which helped shape the design of the interview and survey tools and informed the selection of participants who could reflect on its utility and limitations. Table 2 contains the extraction results in ascending order by year.

**Table 2:** The 21 included papers’ extraction results in ascending order by year.

Authors and year	Topic of Research; Location	Which stages were explicitly reported?	Which principles were explicitly reported?	Applied Leask et al. definition of co-creation	Complementary frameworks used	Explicit critical appraisal of Leask et al. framework
Leask et al. [13] (2019)	Physical Activity Guidelines; the United Kingdom	Evaluating	None	No	None	No
Aaby et al. [14] (2020)	Organizational Health Literacy Responsiveness in Cardiac Rehabilitation; Denmark	Evaluating	Sampling, Manifesting Ownership, Evaluating the process, Evaluating the intervention	No	The OPtimising HEalth LIteracy and Access (Ophelia) approach	No
Riordan et al. [15] (2020)	Diabetic retinopathy screening; Ireland	None	None	Yes	Theoretical Domains Framework; Behaviour change technique, and The Consolidated Framework for Implementation Research	No
Mansson et al. [16] (2020)	Balance and leg strength; Sweden	Planning and Reporting	None	No	None	No
van Dijk-de Vries et al. [17] (2020)	The development of a Co-creation Impact Compass; The Netherlands	None	Manifesting Ownership	Yes	The Co-creation Impact Compass	No
Hug et al. [18] (2020)	Pulmonary rehabilitation for people with chronic obstructive pulmonary disease; Australia	Planning, Conducting, Evaluating, and Reporting	Framing the study, Sampling, Defining the Procedure, Manifesting Ownership, Evaluating the process, Evaluating the intervention	No	None	No
Cedstrand et al. [19] (2020)	Organizational and social working conditions in the construction industry; Sweden	None	None	No	A logic model for the intervention and evaluation design	No
Strobl et al. [20] (2020)	Gender-sensitive physical activity programs; Germany	Planning and Evaluating	None	No	None	No
Geelen et al. [21] (2020)	Physical Activity in Adults; The Netherlands	Planning and Conducting	None	No	The Behavioural Change Wheel	No
Latomme et al. [22] (2021)	Physical Activity of fathers and their children; Belgium	None	None	No	Behavioural Change Wheel	No
Merritt et al. [23] (2021)	Interventions to encourage healthy diets; Jordan	None	None	Yes	None	No
Driessen-Willems et al. [24] (2021)	Enhance healthy nutrition; The Netherlands	Evaluating	None	Yes	They developed a conceptual framework based on the previous research	No
Kohler et al. [25] (2021)	Physical activity promotion; Germany	Planning	None	*Yes*	*Developed a six-phase action-oriented framework for community-based Physical Activity promotion*	No
Cedstrand et al. [26] (2021)	Occupational health in the construction industry; Sweden	Evaluating	Evaluating the process, Evaluating the intervention	*Yes*	*The Cocreated program logic (COP) process and the Behaviour Change Wheel*	No
Antwerpes et al. [27] (2022)	Alcohol use disorder; France	None	None	No	None	No
Lundell et al. [28] (2022)	eHealth tool for people with chronic obstructive pulmonary disease; Sweden	None	Manifesting ownership and Upskilling	Yes	None	No
Maaløe et al. [29] (2022)	Guidelines and training to improve childbirth care; Tanzania	None	Manifesting ownership	No	They developed a framework for co-creating and implementing Clinical Practice Guidelines and associated training	No
Grüne et al. [30] (2022)	Physical activity interventions; Germany	Evaluating	None	No	None	No
Vargas et al. [31] (2022)	Healthier food retail environments	Reporting	None	No	None	No
Illiano et al. [32] (2022)	Stimulate physical activity and cognitive function; Belgium	None	Sampling	Yes	Community-based participatory research framework, the Behaviour Change Wheel, the COM-B model, and the Theoretical Domains Framework.	No
Reich et al. [33] (2022)	Mindfulness for youth at risk for psychosis; Australia	None	None	No	None	No

### 3.2 Interview and survey

From the 21 included papers, 15 lead authors met the criteria for interview invitation. However, one author, who was the original developer of the PRODUCES framework, was not invited to avoid over-representation of developer perspectives. Accordingly, 14 lead authors were invited to participate in interviews. Eleven responded to the invitation, and four ultimately completed an interview.

To capture additional perspectives from the remaining included publications, the 16 lead authors who did not participate in interviews were invited to complete an anonymous survey. Nine responses were received. In total, 13 of the 21 included papers were represented through either interview or survey participation, providing empirical input from just over half of the identified studies.

#### 3.2.1 Quantitative analysis results

Both the interviewees and survey respondents (SRs) were asked to give the overall PRODUCES framework a rating from ‘very poor’ to ‘very good.’ Most participants gave it a ‘good’ Rating (10 Interviewees and SRs), and three interviewees and SRs gave it a ‘very good’ Rating.

The 'good' rating was primarily given due to the framework's clarity and usability, though it has some limitations in practical application. Participants found PRODUCES clear, structured, and user-friendly, making it useful for planning and designing co-creation processes. Some highlighted its potential as a checklist to ensure no key aspects are missed. However, they noted gaps in guidance, especially in evaluation and reporting, making it less practical for real-world applications. Some users also felt that while it provides a good starting point, it requires further refinement to enhance usability across different stages of co-creation.

The 'very good' rating was given because participants considered the framework to be comprehensive and provided guidance through all the stages of the co-creation process. The participants appreciated that it offered practical recommendations, noting that they were previously unaware of any other frameworks that provide such detailed, step-by-step support for running a co-creation process effectively.

The participants also rated each of the four stages and six principles, expressing their overall satisfaction with each. Table 3, Table 4 visualise their response and rating of the stages and principles. Overall, the most liked stages were the *Planning* and *Reporting stages*. The least liked were the *Conducting* and *Evaluating* stages. The most liked principles were *Sampling, Manifesting Ownership,* and *Evaluating the process*. The least liked were *Framing the Study*, *Defining the Procedure,* and *Evaluating the Intervention*.

**Table 3:** Satisfaction rating for the four stages (survey responses (n = 9) and interview responses (n = 4)); n = number of participants.

Satisfaction rating	PLANNING	CONDUCTING	EVALUATING	REPORTING
**Very dissatisfied**	0%	0%	0%	0%
**Dissatisfied**	11.1% (n = 1)	0%	10% (n = 1)	12.5% (n = 1)
**Neutral**	22.2% (n = 2)	25% (n = 2)	10% (n = 1)	12.5% (n = 1)
**Satisfied**	11.1% (n = 1)	50% (n = 4)	50% (n = 5)	37.5% (n = 3)
**Very satisfied**	55.6% (n = 5)	25% (n = 2)	30% (n = 3)	37.5% (n = 3)

**Table 4:** Satisfaction rating for the six principles (survey responses (n = 9) and interview responses (n = 4)); n = number of participants.

Satisfaction rating	Framing the Study	Sampling	Defining the procedure	Manifesting Ownership	Evaluating the process	Evaluating the intervention
**Very dissatisfied**	0%	0%	0%	7.7% (n = 1)	0%	0%
**Dissatisfied**	0%	0%	0%	25% (n = 2)	11.1% (n = 1)	0%
**Neutral**	16.7% (n = 1)	28.6% (n = 2)	0%	25% (n = 2)	11.1% (n = 1)	0%
**Satisfied**	50% (n = 3)	28.6% (n = 2)	66.7% (n = 4)	12.5% (n = 1)	33.4% (n = 3)	85.7% (n = 6)
**Very satisfied**	33.3% (n = 2)	42.8% (n = 3)	33.3% (n = 2)	37.5% (n = 3)	44.4% (n = 4)	14.3% (n = 1)

#### 3.2.2 Qualitative analysis results

**Fig. 3:**
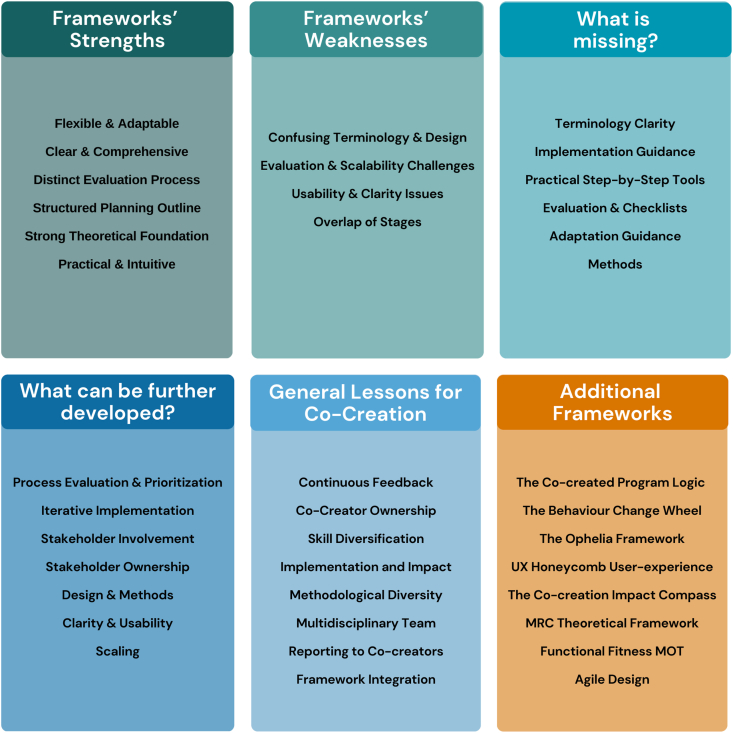
Key Results from the interview and survey responses, ranging from framework strengths and weaknesses to suggested changes, general lessons learned about co-creation, and additional frameworks to complement PRODUCES.

The following are the coded results from both the interviews and the survey, and the codebook is in Supplementary File 3. Fig. 3 visualizes the key findings from the qualitative data sourced from the interviews and surveys.

#### 3.2.3 Frameworks'strengths

Overall, the framework is praised for being flexible and broad, offering a clear, intuitive, and comprehensive structure that adapts to various co-creation contexts and covers all key aspects, from defining what to co-create to guiding practical planning and evaluation. Both interviewees (I) and survey respondents (SRs) highlighted its strength.

The guideline's strengths lie in the fact that it is a “*flexible and broad*” approach (I1) and, in being so it “*could be adapted to the situation [and] leave room for flexibility*” (I3). It has been said to be “*well-designed*” (I2) and “*comprehensive, covering a lot of things*” (I3). I4 appreciated that “*it shows everything important*,” and multiple SRs highlighted that it “*covers all the main areas*.”

I1 appreciated “*the clarity on what to co-create (the aim, to be specific)*” together with I2, who believes the framework “*is built in a way that is easy to understand, it's intuitive.*” Similarly, the SRs noted that the framework is “*clear and user friendly*” (SR3) and described it as “*clear, easy to use, very comprehensive*” (SR2).

I1 and I2 appreciated the suggestion to evaluate both the process and the intervention. Similarly, Interviewee 2 believed that the “*PRODUCES’ way of describing evaluation* supported *the line of thinking*.” I1 enjoyed the clear evaluation distinction between the effectiveness and the process, and the sampling section and scalability mention of the framework. Likewise, I3 said, “*I agree with the evaluation principles. In our paper, we only evaluated the intervention, but in the thesis, we discuss more about how we say the process of doing the co-creation*.” SR8 also comments, “*I like the clarity given to the difference between process and outcome evaluation*.”

Similarly, I2 said “*some of the elements, headline, and structure very much correspond to what I did do*” and appreciated “*the way it [the framework] goes through problem, objective, design is how you would naturally plan and work with your projec*t.” SR7 observed, “*as a model, it looks like a good planning tool for a participatory action research initiative*.” SR6 adds that PRODUCES “*can be used as a kind of checklist to make sure you don't miss any important aspect*.”

The PRODUCES framework helped I2 “*argue that it [the co-creation] had some theoretical foundation”* while I3 expressed that the framework is “*well explained as it includes examples from different areas*.” SR2 also remarks on its uniqueness by stating that they were previously unaware of any frameworks that provided such practical recommendations for running a co-creation process. Likewise, SR5 states, “*for the first time, it offers a framework specifically for co-creation in public health*.”

#### 3.2.4 Frameworks’ weaknesses

The survey and interview responses underline how the PRODUCES framework's design and terminology are confusing, its evaluation and scalability components are insufficiently developed, and the boundaries between different stages are blurred, ultimately impacting its overall usability and practical value.

I1 mentions the difficulty in understanding “*the difference between [Participatory Action Research], [Action Research]*” and found that “*what is confusing is the design in the PRODUCES*” and dealing with “*different stages and principles*.” SR8 questions the rationale behind key elements, implying that certain design aspects (like the emphasis on ownership) are not clearly justified. Likewise, SR9 observes that they “*can't fully use it now to plan*,” highlighting practical design challenges.

I2 believed that “*the last points on the evaluation and scalability are the most difficult ones to use … you may need more than what is in the framework*.” SR9 remarked that there is “*too much focus on evaluating the outcome, the intervention/solution, not on the process itself*” and noted that the “*reporting checklist in [the] paper now [does not have] great usability*.” The same respondent also mentioned the framework is “*too vague perhaps, [and needs] far more theory behind it*.”

I4 specified the following, “*I don't think that conducting [stage] makes sense because that's also part of the planning stage. This is because research does not plan everything, and then they start to conduct a study. But it is developing something together with other people and other organisations. Conducting is in the planning and even evaluation as well*.” Similarly, SR9 indicated a “*mismatch for using this as a planning guide and to facilitate structured reporting*.”

SR8 stated, “*I did not give it [a] very good [rating] because the reporting section was a little confusing initially*,” and questioned why certain elements like ownership aren't integrated as checklist items. SR6 adds that while important, some aspects are “*a bit difficult to grasp*.” Additional comments from SR9 suggested that the framework “*needs to be used and improved*” to enhance its practical applicability.

#### 3.2.5 Needs: what is missing

This section highlights areas that were missing from the framework. The survey and interview responses highlighted the need for clearer introductory guidance on key concepts, along with practical, step-by-step tools for planning, implementing, and evaluating co-creation. As well as adaptable frameworks that account for both intended and unintended outcomes and support context-specific applications.

Several participants stressed the value of an introductory section to clarify terminology. I3 stressed the importance of an introductory section that includes “a *brief overview of the terminology*” related to participatory approaches, such as co-creation, co-design, and participatory action research.

Guidance on implementation strategies was another key gap. I1 noted a lack of direction on modifying implementation strategies when needed and integrating co-creation with other tools and advocated for further “*implementation guidance*.” Similarly, SR5 and SR7 requested “*practical step-by-step guidance*” and methods that could help structure the planning and evaluation process, and SR9 noted that the framework “*doesn't help enough with how to actually plan those things, how to go about them*.”

There was a strong call for structured evaluation criteria and practical tools to support co-creation efforts. SR8 suggested creating a checklist “*similar to PRISMA*” [34] to guide planning and reporting. SR7 emphasised the need for a “*guide to know what to do with the evaluation*,” while I1 stressed the importance of considering “*unintended consequences [and] outcomes you did not plan for*.”

Finally, participants also stressed the need for greater adaptability. SR5 questioned whether problem identification should be co-created, advocating for a more flexible approach. SR3 noted a need for a “*modified version for adaptation of co-created interventions*” to address different contexts. I4 reinforced this point, stating that guidance should focus on “*making the guidance more practical for your readers*.”

#### 3.2.6 Needs: what can be further developed

This section discusses areas that were present in the framework but needed to be expanded on and developed further. Survey and interview responses emphasised the need for more comprehensive, practical guidance throughout the co-creation process, particularly in design, implementation, stakeholder involvement, and evaluation to ensure adaptability across different contexts.

Participants highlighted the need for clearer explanations of design and methodology. I1 wished for guidance to “*elaborate on the design, methodologies and how this affects the research*,” particularly during the conducting stage. Similarly, SR2 and SR1 requested “*greater explanation around the 'design' element*” and “*more on scaling*.” SR9 further emphasised, “*Perhaps more guidance on how to actually build the 'procedure'; how to decide what that will look like.*”

While the framework emphasises iteration, I1 noted that it mainly focuses on iteration until an intervention is defined, whereas “*when you start the implementation, you also need to feed it back and change it again*.” SR5 supported this, calling for more guidance on how to follow up on co-creators' experiences to enhance engagement and ownership.

Ensuring co-creator ownership was a recurring concern. I1 sought further detail on “*who should be involved*” and whether co-creation should involve one group or multiple groups. SR7 highlighted the importance of “*identification of motivations, communication and role assignment that could lead to ownership by the community*.” SR6 similarly noted that “*more information about ownership [is needed], as it was a bit difficult to grasp*.”

In terms of methods, I3 stressed the need for guidance on how to apply co-creation in different settings, suggesting a list of methods: “*We were using the think-aloud method, video recording … there are so many methods.*” I2 emphasised the need for guidance on methods for prioritising with stakeholders, asking, “*How do you prioritise with stakeholders? Do you make your plan about participation with a participation game?*” Similarly, SR2 noted the need for clearer details on identifying problems, prioritising objectives, and sampling methods.

Several participants called for better clarity on process and outcome evaluation. SR5 noted that evaluation in the framework is “*too broad and further guidance is required specifically for co-creation*,” while SR8 suggested “*more clarity about how to use [the reporting] stage and how to incorporate it into a checklist.*”

SR5 and SR8 both highlighted that clarity and usability could be improved, while SR4 remarked that the framework is “useful for research and practice” but could further enhance its practical value with clearer guidance.

#### 3.2.7 General lessons for co-creation

Key lessons gathered in the interviews highlighted the importance of continuous feedback, co-creator ownership, multidisciplinary teams, and impact-driven dissemination. Quotes related to these key insights are depicted in Table 5.

**Table 5:** Key quotes related to general lessons for co-creation, grouped by related topics.

Interviewee	Topic	Quote
Interviewee 1	Continuous feedback	They “*decided we would continuously feed back the results*.”
Interviewee 1	Co-creator ownership	“*Give [the co-creators] the opportunity to decide as much as possible*.”
Interviewee 1	Co-creator ownership	Co-creators “*clearly felt the ownership, but the other problem is that when you co-create in a [omitted setting for anonymity] setting, sometimes they don't take the ownership because they have too many problems*.”
Interviewee 1	Skill diversification	“*You need to have other skills than being a researcher … it is very difficult to teach everything in one framework*.”
Interviewee 1	Implementation and impact	That “*mostly to enhance the implementation – you have to co-create otherwise it's not going to work – but we need to prove that*.”
Interviewee 2	Implementation and impact	It was valuable to “*look at the results, but also the experience of the co-creators*.”
Interviewee 4	Implementation and impact	Recommends the “*need to formulate an idea about your impact. What do we want to do? And about the dissemination of scalability. So that's included in the impact*.”
Interviewee 3	Methodological diversity	“*We threw everything in and did a bit of everything as we had a smaller group*.”
Interviewee 3	Multidisciplinary team	It was important for them to “*build a multidisciplinary team.*”
Interviewee 3	Reporting to co-creators	When reporting, “*we often think about reporting to our research community when we write our paper – trying to describe our work, but it's also about reporting back to the co-creators. I wrote a one-pager, which I sent out to them when I was doing my thesis. It was a nice way to reconnect with them*.”
Interviewee 4	Framework integration	Recommended using the framework in combination with the [co-creation impact] compass-developed guidance

#### 3.2.8 Additional frameworks

Through the interviews and the survey, participants suggested the following additional frameworks and models, used as additional materials to guide their co-creation process to fit a certain context of the field of research, namely: a) The Cocreated program logic (COP) process [35] (I1); the Behaviour Change Wheel [36] (I1 and SR5); the COM-B model [37] (I1); The Ophelia Framework [38] (I2); Agile Design [39] (I2); UX Honeycomb User-experience Model [40] (I2); the Co-creation Impact Compass [17] (I4); Medical Research Council (MRC) Theoretical Framework [41] (SR1 and SR5); and Functional Fitness MOT [42] (SR2).

### 3.3 PRODUCES+ Guideline

Based on the findings from the included studies, in-depth interviews, and survey responses, and with input from co-authors and the original PRODUCES authors, the PRODUCES+ Guideline was created. The updated guideline provides step-by-step guidance, methods, and tools for each stage and principle, supported by relevant publications and resources.

The guideline retained the core strengths of PRODUCES while addressing identified gaps through revised terminology, clearer distinctions between co-creation stages, enhanced practical guidance, and an expanded evaluation component. Table 6 summarises key findings from the evidence synthesis and how these informed revisions to PRODUCES. The updated guideline provides step-by-step guidance, methods, and tools for each stage and principle, supported by relevant publications and resources.

**Table 6:** Mapping the key findings of this study and how it was incorporated into the PRODUCES+ Guideline.

Theme identified through evidence synthesis	Key finding from evidence synthesis	Corresponding revision in PRODUCES+
Framework strengths	PRODUCES was considered flexible, broad, comprehensive, clear, and intuitive. Participants valued its structured approach to planning co-creation, its distinction between process and outcome evaluation, and its usefulness as a planning and reporting tool.	Retained the core structure, principles, and planning approach of PRODUCES while enhancing clarity, usability, and practical application.
Framework weaknesses	Participants identified challenges related to unclear terminology, insufficient distinction between co-creation stages, limited guidance on evaluation and scalability, and difficulty applying some elements in practice.	Refined terminology, clarified distinctions between co-creation stages, expanded guidance on evaluation and scalability, and improved the overall structure and usability of the guideline.
What is missing?	Participants highlighted the need for introductory guidance on co-creation terminology, practical step-by-step guidance, methods and tools, clearer evaluation support, and adaptable approaches for different contexts.	Added introductory definitions, step-by-step guidance, practical methods and tools, expanded evaluation guidance, and additional guidance on adaptation.
What can be further developed?	Further development was needed around design processes, implementation strategies, stakeholder involvement, ownership, methods selection, iteration, and evaluation of co-creation processes.	Expanded guidance on designing and implementing co-creation processes, involving stakeholders, supporting ownership, selecting methods, maintaining iterative processes, and evaluating co-creation.
Additional frameworks	Participants identified complementary frameworks and models that could support specific aspects of co-creation, including implementation, behaviour change, evaluation, adaptation, and user experience.	Added signposting to complementary frameworks and resources to support users in selecting additional approaches according to their context and needs.
General lessons for co-creation	Participants highlighted the importance of continuous feedback, co-creator ownership, multidisciplinary teams, and impact-oriented dissemination.	Incorporated considerations related to ongoing engagement, ownership, and practical implementation throughout the guideline where relevant.

Given ongoing variation in participatory terminology, PRODUCES+ provides an overview of definitions of co-creation, co-design, and co-production from the underpinning literature, while positioning co-creation as the overarching approach guiding the framework. The full guideline is available in Supplementary File 4.

While PRODUCES+ represents an improved and more user-friendly iteration of the original framework, it should be considered an evolving guideline requiring further empirical evaluation to assess its applicability and effectiveness across diverse contexts.

## 4 Discussion

This study addressed the current gaps and weaknesses when executing co-creation in public health by revising and expanding the original PRODUCES framework into an evidence- and user-informed guideline.

Drawing on evidence from a snowballing review, semi-structured interviews, and survey responses, the study generated concrete recommendations, practical resources, and step-by-step guidance designed to guide the user in planning and executing co-creation processes effectively. The guideline builds on the strengths of its predecessor framework, while addressing identified gaps.

### 4.1 Future development

As co-creation continues to evolve, regular updates will be essential to maintain the PRODUCES+ Guideline's relevance across diverse fields within public health and global health. Further empirical evaluation is needed to assess its usability, applicability, and contribution to supporting co-creation practice in varied real-world contexts, including health intervention design, community initiatives, and interdisciplinary collaborations. A future validation phase could invite past participants and future users to review and refine the PRODUCES+ Guideline through methods such as a Delphi survey, helping to strengthen its utility and credibility across sectors.

Terminology inconsistencies remain a challenge in participatory methodologies [1,43]. By accommodating co-creation, co-design, and co-production approaches, PRODUCES+ aims to support broad applicability across contexts. Further integration with complementary frameworks, such as the Behaviour Change Wheel and other established approaches, also presents an opportunity to strengthen collaborative methodologies and support more holistic applications of co-creation.

### 4.2 Limitations

The empirical inputs were informed by individuals with direct experience of the PRODUCES framework, which was appropriate for identifying its strengths, gaps, and opportunities for improvement. However, as participants were identified through publications citing the framework and voluntary survey participation, perspectives from individuals with greater engagement or familiarity with PRODUCES may be overrepresented.

Since the snowballing search was conducted using PubMed Central, it may have excluded relevant studies published in journals not indexed in this database, potentially limiting the comprehensiveness of the included sample.

Additionally, one included publication was authored by a co-developer of the framework; however, this individual did not contribute to the interview or survey components of the study. Additionally, the revision process drew on multiple evidence sources, including published applications, interviews, and survey responses, to incorporate perspectives from different experiences with PRODUCES.

### 4.3 Conclusions

The PRODUCES+ Guideline represents a significant step forward in providing clear, comprehensive, and user-friendly guidance for planning co-creation in public health. It offers practical guidance on how to plan and conduct co-creation in the context of public health. By integrating results from a snowballing review, interviews, and surveys with key informants, it represents a valuable resource for advancing evidence-based practices in co-creation.

## Ethical statement

Ethical approval was obtained from the Research Ethics Committee of the Universitat Ramon Llull in December 2022 (reference number CER URL_2022_2023_009), and all participants provided informed consent.

## Author contributions

DMA and GRL conceived the study and conducted the snowballing review, data extraction, and development of the interview guide and survey questions. DMA, GRL, MGG, SC, DAS, and CFL collaborated on the coding framework. DMA and GRL recruited participants, conducted interviews, and analysed both interview and survey responses. CFL participated as a test interviewee. DMA and GRL wrote the PRODUCES+ Guideline based on the study results. DMA and GRL drafted the manuscript, and all authors critically reviewed and approved the final version.

## Declaration of generative AI and AI-assisted technologies in the manuscript preparation process

During the preparation of this work, the authors used ChatGPT for brainstorming on how to structure the tables in this manuscript, as well as providing suggestions for revising some of the text to make it more concise. The authors reviewed and edited the output as needed and take full responsibility for the content of the published article.

## Funding

This study has been funded by the European Union’s 10.13039/501100007601Horizon 2020 Research and Innovation Programme under the Marie Skłodowska-Curie grant agreement n° 956501. The views expressed in this paper are the author's views and do not necessarily reflect those of the funders.

## Declaration of competing interest

The authors declare that they have no known competing financial interests or personal relationships that could have appeared to influence the work reported in this paper.
