# Supplementary material for: PRODUCES+: Guidance for co-creation in public health informed by evidence and user experience
Source: Public Health Pract (Oxf). 2026 Jul 8;12:100825. doi: 10.1016/j.puhip.2026.100825 (PMC13382120; doi:10.1016/j.puhip.2026.100825)
Supplement: Multimedia component 1 [file mmc1.pdf]

Supplementary File 1. Interview Guide

For included authors

Date:
Interviewer:
Interviewee:
Note-taker:
Interviewee's reference paper:
Link to Mentimeter:
https://www.mentimeter.com/app/presentation/algfdznizen1z6sgfoh3uuz9zwnjwyex

Interview Framing:

A quick round of introductions, and re-confirm they are comfortable with this interview being recorded.

We are assessing the PRODUCES framework's **"practicality",** defined as *understandability and clarity of key constructs, ease of use and comprehensiveness in terms of coverage of adaptation and evaluation recommendations* (Lobczowska et al. 2022).

Introduce what we mean by PRODUCES framework, and the paper where we found their co-creation project where they applied all or part of PRODUCES.

Semi-structured Interview Guide:

Introduction:

Opening questions:

1. **Please describe your experience using the PRODUCES (Leask et al. 2019) framework.**

2. **Please rate the overall quality of the PRODUCES Framework.**
    1. very poor
    2. poor
    3. acceptable
    4. good
    5. very good

3. **Why did you give it that rating? (*layout, clarity, usability, etc.)***

4. **We noticed you used the [insert name of framework] framework in your study, why did you feel you needed another framework beyond PRODUCES?** (optional question if the participant used an additional framework).

<u>**Overall Framework - I like, I wish, I wonder:**</u>

5. **<u>I like:</u> What do you like about the framework/paper?**

6. **<u>I wish:</u> What do you wish was different about the framework/paper?**

7. **<u>I wonder:</u> What would you like to see in a new version of the framework/paper? (*e.g. what were you lacking in terms of guidance; usability or format; suggested methods; implementation process, etc.*)**

<u>**The Four Stages:**</u>

We want to discuss the clarity and usability of the framework, by assessing the stages and principles separately. Ask these questions using MentiMeter, and the note-taker can input their responses.

We noticed we mentioned [*mention any stage they used*] in your paper. Can you answer the following question, to the best of your knowledge?

8. **How satisfied were you with the description and guidance about the [*insert name of stage they used*] stage:**
    - Strongly dissatisfied.
    - Dissatisfied
    - Neither agree nor disagree
    - Satisfied
    - Very satisfied
    - No opinion
9. **Why did you select [*state their answer to #8*]?**

(Repeat questions 8-9 for every stage that was mentioned in their study).

10. **Is there a reason you did not use the [*insert names of stages they did not use*] stages?**

11. **Did you pull any stages from other sources to complement PRODUCES? Other frameworks/ sources?**

<u>The Five Principles:</u>

We noticed we mentioned a [*insert number of principles mentioned*] principles in your papers. Can you answer the following question, to the best of your knowledge?

12. **How satisfied were you with the description and guidance about the [***insert name of principle they used]* **principle:**
    - Strongly dissatisfied
    - Dissatisfied
    - Neither agree or disagree
    - Satisfied
    - Very satisfied
    - No opinion
13. **Why did you select [***state their answer to #12***]?**

(Repeat questions 12-13 for every principle that was mentioned in their study).

14. **Is there a reason you did not use the [***insert names of principles they did not use***] principles?**

15. **Did you pull any principles from other sources to complement PRODUCES? Other frameworks/ sources?**

<u>Closing:</u>

16. **Do you have anything else to add?**

References:

Lobczowska, K., Banik, A., Romaniuk, P. *et al.* Frameworks for implementation of policies promoting healthy nutrition and physically active lifestyle: systematic review. *Int J Behav Nutr Phys Act* **19**, 16 (2022). https://doi.org/10.1186/s12966-021-01242-4
