# Supplementary material for: PRODUCES+: Guidance for co-creation in public health informed by evidence and user experience
Source: Public Health Pract (Oxf). 2026 Jul 8;12:100825. doi: 10.1016/j.puhip.2026.100825 (PMC13382120; doi:10.1016/j.puhip.2026.100825)
Supplement: Multimedia component 2 [file mmc2.pdf]

Supplementary File 2. PRODUCES Revision Survey Guide

For included authors

Hello and thank you for taking the time to respond to this survey. We are assessing the Leask et al. 2019 PRODUCES framework's "practicality", defined as understandability and clarity of key constructs, ease of use and comprehensiveness in terms of coverage of adaptation and evaluation recommendations (Lobczowska et al. 2022).

Please note: If you are unsure as to how to respond, please write "no response" or select "no opinion."

If you have any questions or concerns, feel free to reach out to Giuliana Longworth: giulianaraffaellal@blanquerna.url.edu

* Indicates required question

1. Please describe your experience using the PRODUCES (Leask et al. 2019) framework.*
2. Please rate the overall quality of the PRODUCES Framework?*
   Mark only one oval.

   - ◯ Very poor
   - ◯ Poor
   - ◯ Acceptable
   - ◯ Good
   - ◯ Very good

3. Why did you give it that rating? (layout, clarity, usability, etc.)*
4. Did you complement the PRODUCES framework with any other frameworks or models? If yes, please name them and why:*

Overall Framework - I like, I wish, I wonder:

5. I like: What do you like about the framework?*
6. I wish: What do you wish was different about the framework?*
7. I wonder: What would you like to see in a new version of the framework? (e.g. what were you lacking in terms of guidance; usability or format; suggested methods; implementation process, etc.)*

The Four Stages:

We want to discuss the clarity and usability of the framework by assessing the stages separately. Please rate each stage on a satisfaction scale of 1-5. If you did not use one of the stages in your co-creation project, then please skip the related question.

8. How satisfied were you with the description and guidance about the PLANNING stage:

	1	2	3	4	5	
Strongly dissatisfied	○	○	○	○	○	Very satisfied

9. Why did you give it that rating? (layout, clarity, usability, etc.)
10. What would make you more satisfied with the stage?

11. How satisfied were you with the description and guidance about the CONDUCTING stage:

	1	2	3	4	5	
Strongly dissatisfied	○	○	○	○	○	Very satisfied

12. Why did you give it that rating? (layout, clarity, usability, etc.)
13. What would make you more satisfied with the stage?

14. How satisfied were you with the description and guidance about the EVALUATING stage:

	1	2	3	4	5	
Strongly dissatisfied	○	○	○	○	○	Very satisfied

15. Why did you give it that rating? (layout, clarity, usability, etc.)
16. What would make you more satisfied with the stage?

17. How satisfied were you with the description and guidance about the REPORTING stage:

	1	2	3	4	5	
Strongly dissatisfied	○	○	○	○	○	Very satisfied

18. Why did you give it that rating? (layout, clarity, usability, etc.)
19. What would make you more satisfied with the stage?

20. Did you pull any stages from other sources to complement PRODUCES?

The Five Principles:

We want to discuss the clarity and usability of the framework by assessing the principles separately. Please rate each principle on a satisfaction scale of 1-5. If you did not use one of the principles in your co-creation project, then please skip the related question.

21. How satisfied were you with the description and guidance about the "Framing the study" principle:

	1	2	3	4	5	
Strongly dissatisfied	○	○	○	○	○	Very satisfied

22. Why did you give it that rating? (layout, clarity, usability, etc.)
23. What would make you more satisfied with the principle?

24. How satisfied were you with the description and guidance about the "Sampling" principle:

	1	2	3	4	5	
Strongly dissatisfied	○	○	○	○	○	Very satisfied

25. Why did you give it that rating? (layout, clarity, usability, etc.)
26. What would make you more satisfied with the principle?

27. How satisfied were you with the description and guidance about the "Defining the procedure" principle:

	1	2	3	4	5	
Strongly dissatisfied	○	○	○	○	○	Very satisfied

28. Why did you give it that rating? (layout, clarity, usability, etc.)
29. What would make you more satisfied with the principle?

30. How satisfied were you with the description and guidance about the “Manifesting ownership” principle:

	1	2	3	4	5	
Strongly dissatisfied	○	○	○	○	○	Very satisfied

31. Why did you give it that rating? (layout, clarity, usability, etc.)
32. What would make you more satisfied with the principle?

33. How satisfied were you with the description and guidance about the “Evaluating the process” principle:

	1	2	3	4	5	
Strongly dissatisfied	○	○	○	○	○	Very satisfied

34. Why did you give it that rating? (layout, clarity, usability, etc.)
35. What would make you more satisfied with the principle?

36. How satisfied were you with the description and guidance about the “Evaluating the intervention” principle:

	1	2	3	4	5	
Strongly dissatisfied	○	○	○	○	○	Very satisfied

37. Why did you give it that rating? (layout, clarity, usability, etc.)
38. What would make you more satisfied with the principle?

39. Did you pull any principles from other sources to complete PRODUCES?

40. Do you have anything else to add?

References:

Lobczowska, K., Banik, A., Romaniuk, P. *et al.* Frameworks for implementation of policies promoting healthy nutrition and physically active lifestyle: systematic review. *Int J Behav Nutr Phys Act* **19**, 16 (2022). https://doi.org/10.1186/s12966-021-01242-4
