# Supplementary material for: PRODUCES+: Guidance for co-creation in public health informed by evidence and user experience
Source: Public Health Pract (Oxf). 2026 Jul 8;12:100825. doi: 10.1016/j.puhip.2026.100825 (PMC13382120; doi:10.1016/j.puhip.2026.100825)
Supplement: Multimedia component 3 [file mmc3.pdf]

Supplementary File 3. Qualitative Analysis Codebook

Name	Description	Example Quote	Indicators
Need: What is missing?	Refers to gaps or missing elements of the framework. Could be calling for additional guidance, missing components, or aspects that are not explicitly included.	"Not the process itself, but for the people that took part. You're changing the co-creators minds. Some kind of guidance on how to describe that in a scientific way would be nice."	"could be nice"; "additional"; "missing"; "not included."
Need: What can be further developed?	Highlights areas of the framework that require further elaboration or refinement. Could be calling for more details, or deeper exploration of exciting aspects.	"The iterative process is emphasized but from what I understand it's mostly focusing on the iterative until you reach some type of intervention activity; when you start the implementation you need to feed it back and change it again."	"elaborated"; "more guidance"; "broader"; "not detailed enough"; "go further."
Framework strengths	Recognizes positive aspects of the framework, and could include appreciation for specific elements.	"The sampling was interesting and when I read it now, we did in the same way but I don't remember taking it from here."	"appreciated"; "I like"; "was useful"; "nice"; "makes sense."
Framework weaknesses	Identifies aspects of the framework that are unclear, confusing, or difficult to interpret. Includes feedback on elements, lack of information, or areas that need improvement.	"What is confusing is the design in the PRODUCES.."	"confusing"; "difficult"; "not informative"; "unclear"; "did not like."

Additional Frameworks	Refers to other frameworks relevant to co-creation. Highlights complementary perspectives that could enhance understanding or application.	“co-creation program logic: It is mainly a concept of how to design interventions and what it does is that you design the programme object together. It is highlighting the iterative quality of the process – back and forward.”	N/A
General lessons	Captures reflections on co-creation, including key takeaways, insights gained, and overall impressions.	“I thought it was really fun, interesting and rewarding to do this co-creation work. You get so much from the co-creators.”	“learned”; “we did”; “we used”; “reflection.”
