# Supplementary material for: PRODUCES+: Guidance for co-creation in public health informed by evidence and user experience
Source: Public Health Pract (Oxf). 2026 Jul 8;12:100825. doi: 10.1016/j.puhip.2026.100825 (PMC13382120; doi:10.1016/j.puhip.2026.100825)
Supplement: Multimedia component 4 [file mmc4.pdf]

2025

PRODUCES+: Evidence-based Co-Creation Guideline

HEALTH CASCADE

EVIDENCE BASED CO-CREATION FOR PUBLIC HEALTH

This project has received funding from the European Union's Horizon 2020 research and innovation programme under grant agreement No 956501

By **Danielle M. Agnello** and

Giuliana R. Longworth (co-first authors)

v2.0 March 2025

PRODUCES+

Evidence-based Co-Creation Guideline

HEALTH CASCADE
EVIDENCE BASED CO-CREATION FOR PUBLIC HEALTH

Marie Skłodowska-Curie Innovative Training Network funded by the European Union's Horizon 2020 research and innovation programme under Marie Skłodowska-Curie grant agreement n° 956501

Version 2.0

Public

This project has received funding from the European Union's Horizon 2020 research and innovation programme under grant agreement No 956501

Table of Contents

Table of Contents 2

1. Background 4

2. Introduction 4

Purpose 4

Terminology 4

Scope of this Guideline 6

How was this guideline created? 6

Theory for co-creation 7

Ethics in co-creation 7

Methods for co-creation 8

Implementation and evaluation of co-creation 9

3. Should I co-create? 10

4. Stages of the co-creation process 11

Stage 1: Planning your co-created Intervention 11

Principle 1: Framing the aim of the study 11

Frameworks for Framing the Aim of the Study 12

Method Categories for Framing your Study: 14

Principle 2: Sampling 15

Method Categories for Sampling: 16

Stage 2: Conducting your co-creation 17

Principle 3: Manifesting ownership 17

Methods for Manifesting Ownership: 18

Principle 4: Defining the procedure 18

Method Categories for Defining the Procedure: 18

Stage 3: Evaluating co-creation 21

Principle 5: Evaluating the co-creation process 22
Continuous reflection and iteration 23
Principle 6: Evaluating the co-created intervention 23
Method Categories for Evaluation: 24
Evaluating co-creation methods and process 25
Stage 4: Reporting 26
Reporting Template 26
Method for reporting: 26
Scaling a co-created intervention 26
Adapting a co-created intervention 28
7. Conclusion 29
Annexe 1: PRODUCES+ Planning or Reporting Template 30
References 36

1. Background

The use of co-creation in many fields of science has grown exponentially in recent years. It is also now expected, and often demanded by funders, governments, and policymakers to democratise and accelerate the impact of research and projects. Many claims about the benefits of co-creation have been made but surprisingly there is a total lack of research investigating the validity of these claims and quantifying the actual impact of co-creation on public health[1].

Health CASCADE is the first multidisciplinary expert network dedicated to researching the methodology of evidence-based co-creation for public health and health promotion. Health CASCADE aims to CASCADE co-creation skills and expertise by training a new community of professionals capable of working across disciplines, and public and private sectors. From 2021 to 2024, a coordinated effort by seven beneficiaries and 14 partner organisations from eight European countries has a multidisciplinary approach in multi-environment and -sectoral contexts to define, test and finalise the concept of evidence-based co-creation for public health.[2]

To respond to the lack of a "precise or systematic framework to plan or develop co-created public health interventions and evaluate their effectiveness and impact"[3] and lack of information and no clear step-by-step guide on how to do this,[4] Health CASCADE worked to develop this document, the PRODUCES+ Guideline, to provide more user-friendly and systematic recommendations and guidance on the planning and conducting co-creation for public health; focusing on how to incorporate theory, ethics, implementation, methods, and evaluation throughout a co-creation process.

2. Introduction

Purpose

This guideline aims to provide researchers and practitioners with guidance and recommendations for planning and implementing co-creation for public health.

The objective of this guideline is to revise and build further on Leask and colleagues' PRODUCES framework by providing relevant resources that can assist with each stage of the co-creation process: planning, conducting, evaluating, and reporting.[3]

Terminology

Definitions of co-creation for public health

There exist multiple definitions of co-creation, and our attempts to converge on a singular definition proved challenging and lacked added value. Consequently, we have chosen to embrace the diversity

of definitions, recognizing that different interpretations of co-creation may serve varying research aims. Specifically for Health CASCADE, several definitions have been used, mainly because of the specific scope of the research. For instance, some studies may adopt a broad definition to ensure comprehensive coverage in a scoping review, while others may adhere to a definition that aligns with their co-creation protocol. As such, we provide below a selection of definitions utilized in peer-reviewed papers:

- **Vargas et al. 2022:** Co-creation refers to the collaborative approach of creative problem-solving between diverse stakeholders at all project stages. It emphasises diverse stakeholders at all parts of an initiative process, beginning with determining and defining the problem through to the final stages of a project.[5]
- **Agnello and Loisel et al. 2023:** Co-creation is any act of collective creativity that involves a broad range of relevant and affected actors in creative problem-solving that aims to produce a desired outcome.[6]
- **Messiha et al. 2023:** Co-creation is defined as an all-encompassing principle of collaboration and innovative problem-solving among various stakeholders across all initiative phases (e.g., from problem identification to evaluation).[7]
- **An et al. 2023:** Co-creation is defined as a branch of participatory research which indicates that 'end-users' can be co-creators whose experiences provide value and innovation.[8]
- **Longworth et al. 2024:** Co-creation is a collaborative approach to developing solutions, such as interventions aimed at enhancing public health, ensuring that these solutions align with the needs and preferences of stakeholders and the target population. It involves engaging with diverse stakeholders at all project stages, from determining and defining the problem through to the final stages of a project.[9]

Definitions of the co-approaches

- **Co-design:** Co-design describes active collaboration between stakeholders in designing solutions to a prespecified problem. It promotes citizen participation to formulate or improve specific concerns (i.e. service or product improvement, better prevention activities, more resources, better-trained health promotion staff and, evidence-informed initiatives). Co-design is not always formally documented and can take the form of group problem-solving, critically, after the problem has been determined.[5]
- **Co-production**: Co-production engages stakeholders in the implementation of a previously agreed solution (strategy) to a previously agreed problem and focuses on how to allocate resources and assets within these constraints, to achieve better outcomes. Co-production occurs after the initiative was designed and takes place at the point of implementing the initiative.[5]

Other relevant definitions

- **Co-creation method**: Co-creation methods encompass a diverse range of tools, activities, approaches, and techniques strategically employed across the entirety of the co-creation process. These methods serve various purposes, including but not limited to data collection, facilitation, recruitment, reflection, data analysis, and dissemination, allowing for flexibility in achieving diverse objectives.[10]
- **Ethics in co-creation:** Seeking to act in virtue throughout the entire process, amongst the existence of divergent interests, power dynamics, and knowledge differences, through intentional behaviour that is conscious of the shared responsibility and commitment to engage in a collective effort directed at improving public health within a specific context, in a professional, justifiable, deliberative manner, while considering mental and physical wellbeing for stakeholders involved.[11]

Scope of this Guideline

Guideline Structure. This guideline for evidence-based co-creation for public health interventions is structured around the stages and principles of a recent seminal publication by Leask et al.[3] Their PRODUCES framework was selected because itis the only peer-reviewed implementation and evaluation framework that was developed for co-creation in public health.

How was this guideline created?

This guideline was developed through a two-part research process conducted by Agnello and Longworth, as detailed in the accompanying publication.

First, a snowballing literature search was performed, starting from papers that cited the Leask et al. 2019 publication.[3] The aim was to identify all articles that reference the PRODUCES framework, ensuring a comprehensive understanding of how the framework has been used and interpreted in the literature.

Second, the researchers conducted in-depth interviews and surveys with users of the PRODUCES framework. This phase aimed to gather critical feedback to assess the framework's strengths, limitations, and practical applications. Insights from this user engagement were used to inform the development of this user-friendly guideline, to support more effective and accessible co-creation practices.

Based on findings from the included studies, interviews, and survey responses, along with input from co-authors and the original PRODUCES authors, this guideline was developed as an updated and more user-friendly iteration of the PRODUCES framework. This version retains the strengths of the original

while addressing key weaknesses by incorporating clearer language, refined distinctions between co-creation stages, and a more structured format. To support practical application, this guideline integrates step-by-step methods and frameworks for each stage and principle, referencing relevant publications and materials. The evaluation component has been expanded to provide more detailed guidance, and new sections on scaling and adaptation reflect emerging research. A comprehensive checklist supports planning and reporting, and the guideline signposts complementary frameworks to enhance usability at different stages of the co-creation process.

Theory for co-creation

Theory-based principles offer a structured foundation for co-creation, helping ensure that practices are evidence-informed and comparable across studies. However, recent research has highlighted a lack of explicit theoretical frameworks in co-creation, co-design, and co-production within public health. For example, Messiha et al. 2024 found only 10 explicitly stated theories across the literature, such as Empowerment Theory, Social Learning Theory, and Narrative Theory, with Empowerment Theory being the most commonly used.[7] To address this gap, the authors proposed critical realism as a promising meta-theoretical foundation to better guide and interpret co-creation research.[12] In parallel, a hybrid review identified key common dimensions of co-creation that could serve as theory-based principles, reinforcing the need for a multi-dimensional approach and a shared language to advance the field.[13]

We encourage users of this guide to review these sources when selecting a theoretical foundation for their co-creation process, as doing so can strengthen both the design and evaluation of their initiatives.

Ethics in co-creation

The following reflections on ethics for co-creation are drawn from Chrifou's 2022 report: *Ethics in co-creation for public health: a preliminary, multi-source perspective.*

Ethical challenges are always present in co-creation, and ethical decisions must be made throughout the process. Ethics involves understanding the value systems that inform human principles and actions. It is concerned with what we ought to do, what we ought to avoid, and how to distinguish between right and wrong behaviour.[11]

In the context of co-creation, ethics extends far beyond procedural requirements such as legal frameworks, informed consent, disciplinary codes of conduct, or ethics review boards. Relying solely on procedural ethics risks overlooking the deeper values that underpin meaningful collaboration.

From a methodological perspective, overstating the level of co-creation while only engaging in a limited partnership with participants can undermine the concept and reduce its credibility over time.[11]

Additionally, according to Health CASCADE, important values to uphold throughout the co-creation process include: inclusivity, plurality, diversity, representation, sensitivity to context, openness, transparency, accountability, integrity, and neutrality.[11] We encourage users of this guideline to reflect carefully on these values and to explore further ways to embed ethical practice in their co-creation work.

Methods for co-creation

Research methods provide a systematic approach to collecting and analysing data, helping to ensure that findings are valid, reliable, and reproducible. Clear documentation of methods enhances understanding, supports replication, and enables others to build on previous work. Choosing appropriate methods also helps to minimise bias and increases the credibility and trustworthiness of results.[10]

Co-creation is used to address complex and wicked challenges. Relying solely on qualitative methods may limit the effectiveness of co-created solutions and risks resulting in a tokenistic form of participation. To support meaningful and rigorous co-creation, a wide range of methods is needed.

Therefore, the methods included in this guideline represent only a small selection of those relevant to co-creation. For a more comprehensive overview, we recommend consulting:

1. Agnello et al. 2024 Multimedia Appendix 4 and 5, which contains a systematic inventory of co-creation methods containing 956 methods from both academic and grey literature.[10]
2. Agnello et al. 2025 Additional File 3, which contains another inventory of 248 co-creation methods sourced from academic literature. This study also contains details on using these methods across different populations, such as children, people living with disabilities, and marginalised groups.[14]

These datasets include a diverse mix of participatory and creative methods, as well as qualitative, quantitative, and mixed methods approaches that have been well documented in co-creation processes.

We encourage users of this guide to explore and experiment with the methods presented here, and to draw on other relevant approaches from the sources above that suit their specific context, even if they are not included in this version of the guideline.

To support your selection of an appropriate method, we at Health CASCADE developed an infographic with some key questions you can ask yourself when considering which method to apply. This decision support tool is depicted in Figure 1.[15] Agnello et al. 2023 also developed a framework called the Co-Creation Rainbow Framework that supports you in assessing the 'level of participation' of each method.[16] We encourage you to use that framework alongside the Methods Selector infographic and the online Co-Creation Method Selector tool (https://ccmethodsselector.lovable.app) to identify the most appropriate method for each step of your co-creation process.

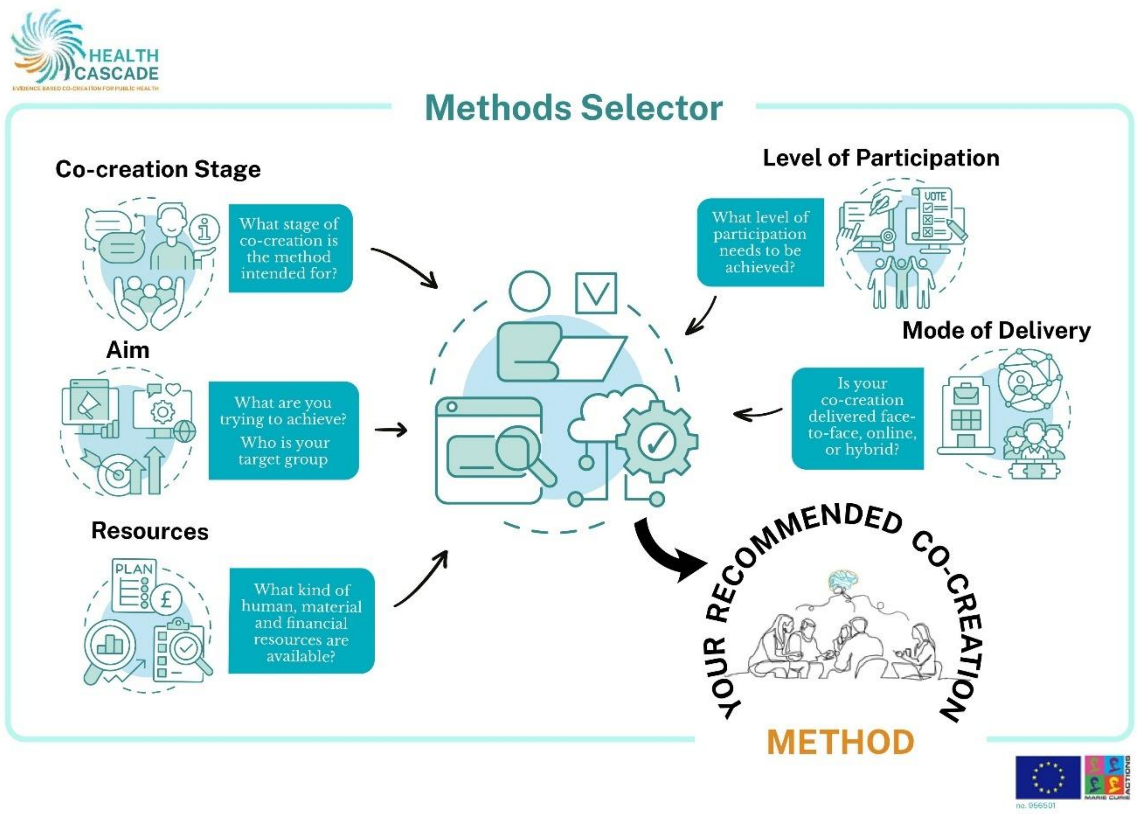

Figure 1. An infographic that can support you in the selection of an appropriate method for your co-creation process (Source: Health CASCADE[15]).

Implementation and evaluation of co-creation

In co-creation, services, activities and interventions are developed in collaboration with intended users and participants. Unlike traditional approaches to implementation research, co-creation seeks to engage with real-world conditions rather than control or eliminate them. This means working directly with the populations who will be affected by an intervention, rather than selecting participants who may not reflect the realities of the target group, such as healthy volunteers.

When interventions are designed collaboratively and grounded in real-world settings from the beginning, the distinction between intervention development and implementation begins to fade. Co-

creation becomes a more dynamic and iterative process compared to linear models of implementation. The 2024 review by Longworth et al. provides a set of recommendations for planning the implementation of co-created interventions, as also represented in Figure 2.[17]

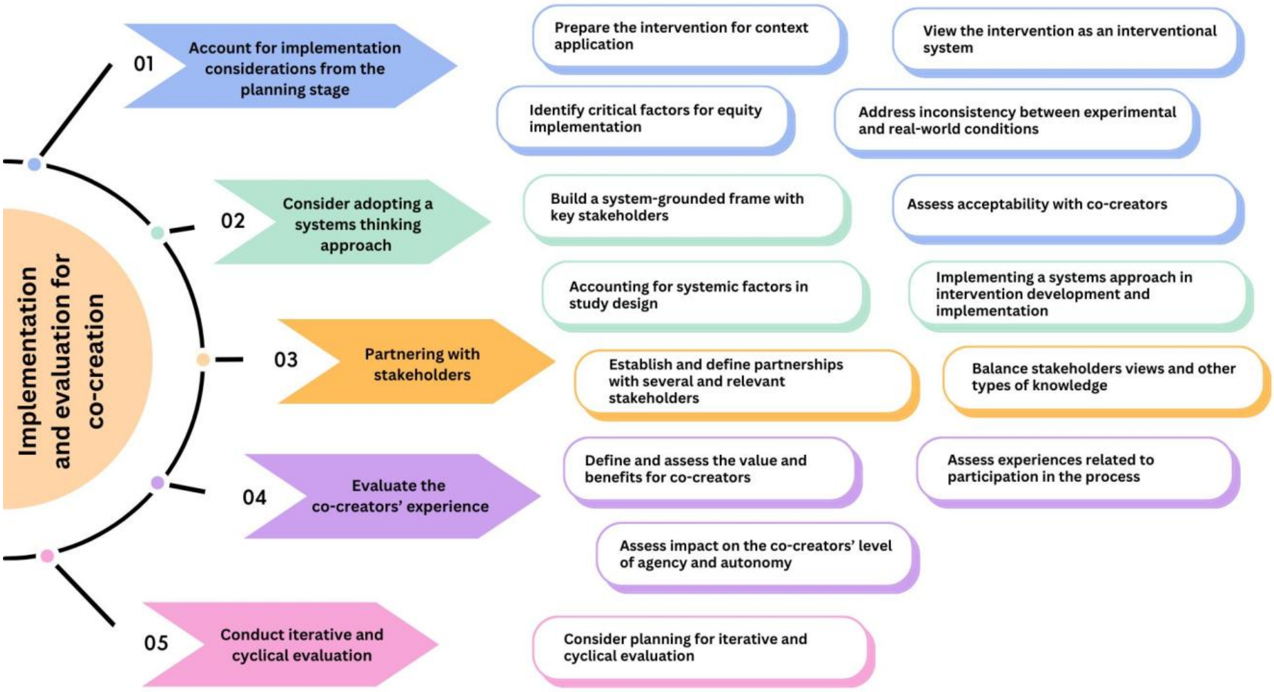

Figure 2. *An infographic with recommendations (Source: Longworth et al.[17]).*

Additionally, if you are interested in applying a systems thinking approach, the recently published DISCOVER framework by Smith et al. 2024 offers planning guidance, implementing and governing co-creation within complex systems.[18]

The implementation and evaluation frameworks included in this guideline are based on a review published by Longworth et al.[17] At present, the only peer-reviewed framework developed specifically for co-creation in public health is the framework by Leask et al.[3] For this reason, the step-by-step guidance provided here draws on their work, alongside relevant methods and frameworks sourced through the Health CASCADE project.

3. Should I co-create?

Co-creation is resource-intensive and requires a lot of time and if applied incorrectly or in the wrong situations, then it can potentially damage collaborator relationships. Therefore, Health CASCADE has created a Decision Support tool to help you decide if co-creation is the correct approach to take. This tool was created iteratively among the co-creation experts in the Health CASCADE project. It can be accessed online and found in Figure 3.[19]

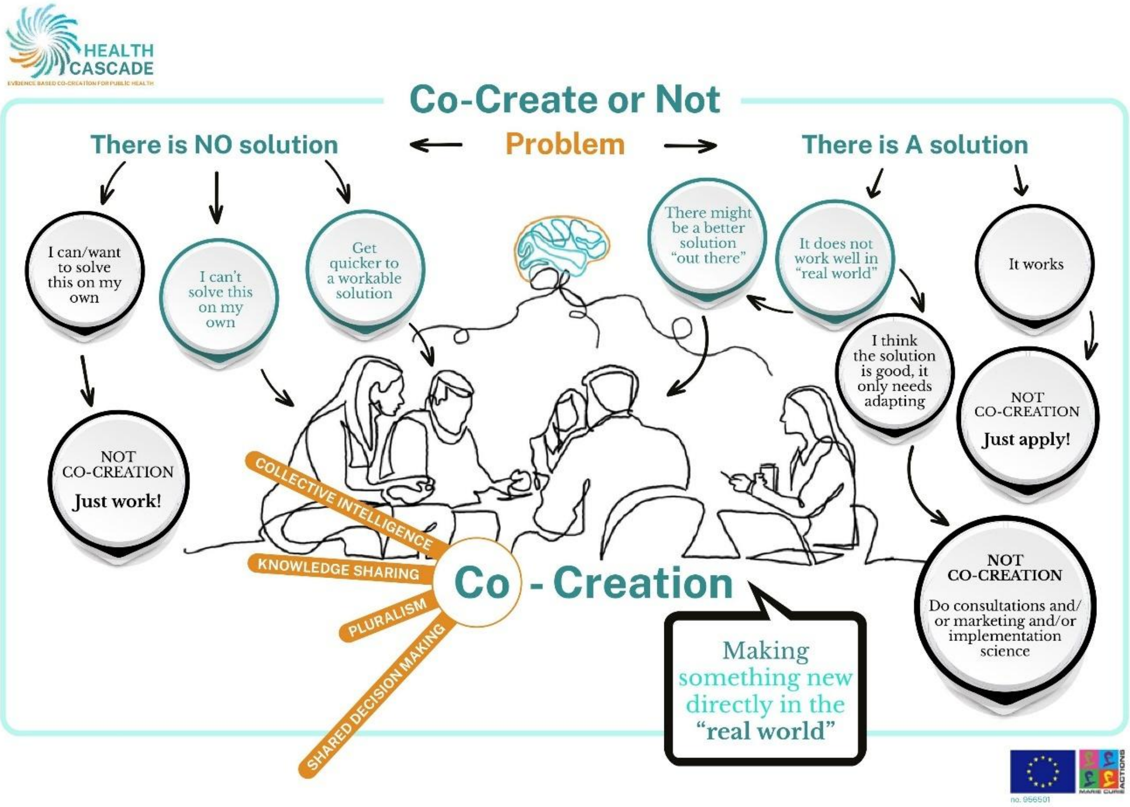

Figure 3. *Co-Creation Decision Support Tree, source: Health CASCADE*

4. Stages of the co-creation process

This section of the guideline goes through the stages of the co-creation process, as described by Leask and colleagues.[3] Each stage includes related principles as well as suggested categories and types of methods.

Stage 1: Planning your co-created Intervention

When academic researchers are initially planning the co-creation, two main principles require consideration: 1) Framing the aim of the Study; and 2) Sampling.

Principle 1: Framing the aim of the study

Framing the aim of the study systematically can help ensure the co-creation process generates trustworthy evidence.

When planning your intervention, remember:

- To ensure transparency with the co-creators about the aim of the process.

- To frame the aim of your study or project in a systematic way to help ensure the process generates scientifically trustworthy evidence.
- To narrow your project aims to increase the success chance of your co-creation process.

- That the non-academic co-creators will influence what the intervention looks like.
- When addressing a complex problem, end-users and other stakeholders may collaborate with academic researchers to define the objectives.
- To narrow to specified health behaviour and population.
- To define what part of the problem the process will address by expressing the specific objective of the process.

- To identify who the co-creators are, as this will be a combination of service providers and end-users.

Frameworks for Framing the Aim of the Study

Frameworks that can help you frame your study:

1. **The PRODUCES framework**[3]: The framework suggests you consider the following elements and ask the following questions when planning your co-creation process:

    - Problem: What is the reason for the process?
    - Objective: What is the aim of the process?
    - Design: What specific participatory methodology is used for co-creation?
    - (End-) Users: Who will use the co-created intervention?
    - Co-creators: Who is engaging in the process?
    - Evaluation: How is success measured?
    - Scaling: How can the co-created intervention be scaled to other contexts?

Frameworks that can help you reflect on the knowledge and evidence used throughout your intervention:

2. **PARIHS framework:** The PARIHS framework values and considers the "quality of the evidence." Grounding an intervention on evidence-based considerations is one of PARIHS' three key constructs. They argue evidence encompasses codified and uncodified sources of knowledge, including research evidence, practitioner experience, community preferences and experiences, and local information.[20] The original article from Kitson and colleagues describes the framework in more detail.[21]

This is particularly relevant to evidence-based co-creation as you want to ensure that diverse forms of knowledge are acknowledged and integrated into the design process, valuing both formal research and lived experience.

Frameworks that can help you reflect upon your intervention context:

1. **The PROCEDE/PRECEED model:** The PRECEDE-PROCEED model is a comprehensive structure for assessing health needs for designing, implementing, and evaluating health promotion and other public health programs to meet those needs. PRECEDE provides the structure for planning a targeted and focused public health program. PROCEED provides the structure for implementing and evaluating the public health program.[22]

2. **TDF (Theoretical Domains Framework):** The TDF is a comprehensive framework a researcher can use to identify factors (i.e., barriers and facilitators) that may influence behaviours and includes considerations around social and environmental factors. It is said to be able to serve as 1) a planning tool to help identify determinants (and appropriate techniques) to address previously identified barriers and facilitators to positively impact outcomes of an implementation strategy; and, 2) as an evaluation tool to assess how effective intervention was at targeting specific behaviour determinants and/or to retrospectively identify factors that contributed to the success and/or failure of an intervention.[23]

   It is said to provide a useful and straightforward guideline on how the TDF framework can be used to assess implementation problems and support intervention design.[24]

Frameworks that can help you choose your facilitation type and methods:

1. **The PARIHS diagnostic and evaluation grid:** The PARIHS framework emphasizes the need for appropriate facilitation to improve the likelihood of success and its diagnostic and evaluative grid has been argued to be useful for choosing the facilitation methods needed.[25] The framework has been argued to be useful "to identify critical evidentiary (e.g., patient experience, information from the local setting) and contextual (e.g., leadership, receptive context) elements that may impact EBP implementation."[26] This framework is intended to support the development of implementation strategies, which heavily rely on facilitation. The PARISH diagnostic and evaluation grip might prove useful when reflecting upon the facilitation methods needed.

Frameworks that can help you define your target behaviour:

1. **The COM-B model:** is part of the Behavioural Change Wheel so it closely aims to support design teams in understanding what will bring about the desired behaviour change in the

target group's Capability, Opportunity and/or Motivation to engage in the behaviour. The framework is highly based on behavioural change theories and beliefs.[27]

Method Categories for Framing your Study:

1. **Assessment:** These methods can be used to further assess your problem or the need for the co-creation process.
    a. Published evidence synthesis: a method for identifying the existence of a problem. It can challenge assumptions and broaden discussions to include data and divergent perspectives.[28]
    b. Needs Assessment: a method for identifying the existence of a problem, or what you will try to achieve with your project.[28]
2. **Establishing the co-creation team:** These methods help you define the type of co-creation team you would like to establish, as well as identify the skills of the co-creators needed to finalize the task.
    a. The 'editorial rights' team / Development team: a group of co-creators that can make final decisions about the intervention. This group should include developers and members of the target population, and it is recommended to include a variety of disciplines and expertise in the team; all members should interact directly with members of the target population.[28]
    b. Establish an action group: this group should be comprised of stakeholders who can use their relevant expertise to build the solution to the problem identified.[28]
    c. Establish a planning group: this group should focus on bringing a variety of stakeholders together, and collaborative working with the target populations and those who will deliver the intervention. This is a method to ensure members of the target population have decision-making rights throughout the co-creation process.[28]
3. **Fieldwork:** A group of methods intended for gaining a deeper understanding of the problem; identify any other factors that can impact the performance of an intervention.
    a. Media portrayal of the co-creation purpose: a method for gathering printed media images, such as from newspapers and magazines, which convey the purpose of the process. Using visual aids as discussion tools in the co-creation process has been identified as an effective tool to enhance the effectiveness of co-creators.[3]

b. Informal discussions w/ people outside the co-creation group: a method to encourage co-creators to informally discuss process topics with non-co-creator stakeholders. It can provide a fresh perspective on the topic and enhance ideas and is an informal form of snowball sampling that may increase the generalisability of findings.[3]

4. **Observation:** Observation methods can be used for obtaining the views of the target population; to help articulate the problem fully or to understand the problem and the context in which the intervention will operate.

   a. Participant observation: a method for using observation of the direct target group is recommended as well as obtaining the views of the target population because people may not be able to articulate the problem fully.[28]

   b. Non-participant observation: a method for understanding the problem and the context in which the intervention will operate. The observation may be of service delivery where the intervention will occur.[28]

5. **Research**: The use of research methods can uncover more vital information about the target population, potential co-creators, past experiences with similar interventions, and needs that may affect the success of the co-creation process and/or the co-created intervention.

   a. Primary Qualitative Research: a method conducted with iterative qualitative research that uses diverse samples and open questions to explore people's experiences and needs. This method uses patients' narratives or archives of patient experiences and observation, consultation with stakeholders, and use of patient and public involvement.[28]

   b. Asset Assessment: a method to determine the strengths of the community in which an intervention will take place.[28]

   c. A systematic review of quantitative evidence of effectiveness: a method to identify what has worked or not worked for a particular intervention. It can be used to identify evidence of the effectiveness of interventions for these problems.[28]

Principle 2: Sampling

The sampling process of recruiting end-users and all necessary expertise as co-creators. The representation of relevant stakeholder groups is dynamic and there are different methods for sampling.[3]

When planning for sampling for co-creators remember:

- Sampling in the co-creation process will be different in scope and nature from linear implementation models, as it selects beneficiaries who may not represent the target population of an intervention but instead intended to build a sampling that is representative of the populations that will be affected by an intervention.
- End-users may be defined specifically or broadly and this might impact the outcome. Defining the end-users, for example, as "older adults" will imply that your co-creation is addressing many other sub-groups while defining them as "ambulatory, community-dwelling older adults" would narrow your target audience.[3]
- End-users' characteristics to take into consideration include age, gender, physical condition, medical history, socioeconomic status, and ethnicity. A representative sample from the end-user group should be recruited as co-creators during the development process and this may include sampling across different characteristics to ensure heterogeneity.[3]
- The sampling for recruiting co-creators is a process with a dual purpose: 1) ensure a representative sample of end-users are recruited as co-creators so the co-created outcome can be utilised by that group or scaled to a population level; 2) ensure there is a representation of all necessary expertise from relevant stakeholder groups.[3]

Method Categories for Sampling:

1. **Mapping:** These methods support gaining a better understanding of the system you may be working within, and different relevant stakeholders, as well as tools for building a theory of change for your co-creation process.
    a. Whole system mapping / participatory systems mapping: this method was developed to respond to complex systems by the production of a causal map of a system that is designed by a diverse set of stakeholders. This method also allows for network analysis with subjective information from stakeholders.[29,30]
    b. Stakeholder mapping: this method can be used to identify the individuals, groups, or organisations affected by a project or who affect its outcomes.[31]
2. **Sampling / Recruitment:** represents a different set of sampling methods for recruiting co-creators, end-users, or the target population. The selection of the specific sampling methods will depend on the aim of your co-creation process.
    a. Convenience sampling: a method to ensure the recruited co-creators are committed and will actively engage in the process. This method is a type of non-probability or non-random sampling where members of the target population that meet certain

practical criteria, such as easy accessibility, geographical proximity, availability at a given time, or willingness to participate are included in the study.[3,32]

b. Maximum variation sampling: a method for finding end-users who cover the spectrum of perspectives, including typical and extreme cases.[3]

c. Opportunistic sampling: a method for recruiting additional stakeholders as co-creators throughout the co-creation process. When using the method, the representation of relevant stakeholder groups is dynamic.[3]

d. Purposeful or Judgement sampling: a method to ensure a representative sample of end-users and relevant stakeholder co-creators are recruited. It is the deliberate choice of a participant due to the qualities the participant possesses. This is a non-random method that does not need underlying theories or a set number of participants. It is typically used in qualitative studies.[3,32]

e. Stratified sampling: a method for sampling across characteristics of interest to create a heterogeneous group with diverse experiences.[3]

Stage 2: Conducting your co-creation

When academic researchers conduct the co-creation, two main principles require consideration: 3) Manifesting Ownership and 4) Defining the Procedure (Leask et al. 2019).

Principle 3: Manifesting ownership

Providing co-creators with a sense of ownership can improve creativity, practice, and knowledge production. Leask and colleagues recommend a set of recommendations for providing ownership, such as *State of ownership*, *Right of ownership*, and *Act of ownership*.[3]

Conduct your intervention by considering the following: Stating your ownership by using the method of *branding the group* and setting out the status of each co-creator at the beginning. You might want to affirm, for instance, that all co-creators have equal standing in the group.[3] Ownership may be acquired incrementally over several workshops through an empowerment process of facilitating openness and perceived control of the process.[3] Key concepts to remember: 1) Defining the right of ownership, and 2) Act upon ownership.

Methods for Manifesting Ownership:

Some methods can be used to manifest ownership throughout the conducting stage.; in alignment with Principle 3, Manifesting Ownership.

1. Branding the group: this method aims to manifest ownership by branding the group.[3]
2. Identifying the rights and responsibilities of the group: this method aims to manifest ownership by outlining group rights and responsibilities. All co-creators have the right and equal status within the group and their responsibility to contribute their ideas.[3]
3. Priority Mapping: assists groups in recognizing strategic goals, metrics, and objectives. This method aids in aligning co-creator views and focusing efforts on addressing mutually agreed-upon priorities.[33].
4. Team purpose and culture method: guides co-creators in defining their collective purpose and collaborative working style through reflection and group discussions, visually representing shared goals and values.[34]

Principle 4: Defining the procedure

Procedural components can be used to facilitate a co-creation process and a protocol for the implementation of the co-creation process is useful to have and guide the process.[3]

Method Categories for Defining the Procedure:

1. **Brainstorming / Mapping:** this currently contains one method that is also a mapping technique, *Concept Mapping*.
    a. Concept Mapping: This method uses a mixed-method approach to enable a group of people to articulate and depict graphically a coherent conceptual framework or model of any topic or issue of interest. It is a mixed method (structured process) designed to enable a group of people to map their ideas for a topic and to articulate and depict graphically a coherent conceptual framework or model of any topic or issue of interest.[35,36]
2. **Categorization Matrix:** this currently contains one method, the *Optimized Honeycomb Model for User Experience*.
    a. Optimized Honeycomb Model for User Experience: this method is a model that comprises qualitative content analysis with a deductive-inductive approach. It's based on the UX Honeycomb model, which is a visualisation tool created by Peter Morville

back in 2004.[37] It is typically used as a teaching tool, to explain the basics of UX (User Experience), but it can also be used as a checklist for designers.[38]

3. **Dialogue:** This represents a group of methods used for generating fruitful and inclusive dialogue with the co-creators and /or target group.

    a. Dialogic Literary Gatherings (DLG): this method is an educational intervention for the collective creation of knowledge and meaning based on the reading of the best literary creations of humanity and the subsequent dialogue between all the participants. Additionally, reading quality literary works increases the capacity to better understand others, facilitating empathy and pro-social behaviour.[39]

    b. Dialogue cafés: this method aims to engage the co-creators in discussions. This method generates new ideas, enables joint decision-making on key strategic issues, discovers new collaboration, and identifies steps for further exploration or implementation. It was created by taking elements from world cafés and dialogue conferences.[40]

    c. Involvement Matrix: this method can be used prospectively to discuss possible roles of patients in different phases of projects, and retrospectively to discuss whether roles were carried out satisfactorily. This method was developed by a co-creation of experienced experts with researchers. Sourced from (Dirk-Wouter et al., 2020).

    d. World cafés: this method follows a principle of good conversation, where all co-creators are given the chance to talk about things that matter to them. This method follows 2 principles: 1) people want to talk together about things that matter to them; 2) if they do, they could create collective power.[40]

4. **Fieldwork:** Some Fieldwork methods are used in the planning stage, but this category contains one method of the conducting stage, *Field Testing of the Prototype*.

    a. Field Testing of the Prototype: this method is also a form of prototyping that allows the end-users to use the prototype in their typical real-world setting to identify any areas for improvement. Therefore, it can also be seen as a type of evaluation method during the conducting stage. The method provides co-creators with an example of what the final intervention may be like and allows them to use the prototype in their typical real-world setting, identify how it could be improved, and will inform the implementation of the intervention itself.[3]

5. **Interview:** These methods are mainly used for evaluating, but some methods can also be used in the conducting stage. These methods aim to source further input from the target group and/or co-creators. The specific type of interview and aim will vary based on the desired outcome.

    a. Semi-structured interviews: this method is an in-depth, qualitative interview where the co-creators are requested to answer pre-set open-ended questions and are utilized extensively in an interviewing format with an individual or sometimes even with a group.[41,42]

    b. Stakeholder interviews: this method is for gathering input from stakeholders relevant to the study; usually open interviews without a particular framework. The interviewees should be treated as deliberative partners rather than research subjects, and rough causal theories about how co-creation was working.[43]

    c. Qualitative interviews: qualitative research interviews are either unstructured, semi-structured, lightly structured, or in-depth. Unstructured interviews are generally suggested in conducting long-term fieldwork and allow respondents to let them express themselves in their ways and pace, with a minimal hold on respondents' responses.[42]

6. **Observation:** Observation methods are mainly used for planning, but this category represents one method that can be used for observation in the conducting stage, *Participant Observation*.

    a. Participant observation: a method for using observation of the direct target group is recommended as well as obtaining the views of the target population because people may not be able to articulate the problem fully.[28]

7. **Prototyping:** This category represents a different type of prototyping method to test your concept and identify any areas for improvement. Prototyping is a complex development task/process where design teams implement ideas into tangible forms. It is important to note that prototypes can change rapidly after stakeholder feedback.

    a. Concept prototyping: this method develops potential concepts to be tested as a prototype. Prototyping is a complex development task/process where design teams implement ideas into tangible forms.[44]

    b. Product prototyping: this is a method for prototyping a physical product or an intervention.[44]

8. **Reflection:** Reflection has been used as a method for research processes and experiences, in ways that recognise the assumptions, frameworks and patterns of thought and behaviour that shape thinking and action. This category contains some methods that can be used for reflecting during the co-creation process.

    a. Toxic River activity: this method creates a process of critically reflecting on the team's success (or failure) in completing the activity/game. The group is asked to reflect on three key themes: 1) setting the agenda, 2) taking people along, and 3) doing it the right(s) way.[45]

    b. Participatory drawing method: this method aims to create a map of the issues that needed to be addressed. Participatory drawing can allow participants to express themselves in alternative ways that do not prioritise or privilege the written or spoken word.[45]

    c. Video recordings: this method provides an unstructured space where the co-creator can video record stories and ideas in response to the various module topics.[45]

9. **Storytelling:** This category of methods aims to capture different voices and empower participants, as well as document and reflect reality. It is also a way of representing individual narratives.

    a. Digital story-making / stories: this is a method for representing individual narratives. It uses a 3–5-minute visual narrative synthesizing images, video, and audio recordings of voice, music, and text to create compelling accounts of experience.[46]

    b. Photovoice: this method aims to empower co-creators, who are often from socially marginalized groups and are unable to articulate their thoughts appropriately into words. By utilising photographs taken and selected by participants, respondents can reflect upon and explore the reasons, emotions and experiences that have guided their chosen images.[47]

Stage 3: Evaluating co-creation

When the core co-creation team are evaluating a co-created intervention, two main principles require consideration: 5) Evaluating the co-creation process (Principle 5), i.e., when assessing the co-creation that led to the intervention development, and 6) Evaluating the co-created intervention (Principle 6), i.e. when analysing the implementation of the co-created intervention.

Principle 5: Evaluating the co-creation process

To address this gap in the literature, the authors of this guidance developed the PROSECO framework (PROcesS Evaluation framework for CO-creation in Public Health) to guide the evaluation of co-creation processes in public health interventions. Using a multi-step, iterative approach, including two scoping reviews, expert input, and design refinement, the study identified 37 evaluation components across five key dimensions: Delivery, Participation, Experiential, Context, and Impact.

The PROSECO framework provides a flexible, evidence-based set of evaluation components to support the assessment of co-creation processes and methods in public health. A full list of the components shown in Figure 4 are provided in the paper [48].

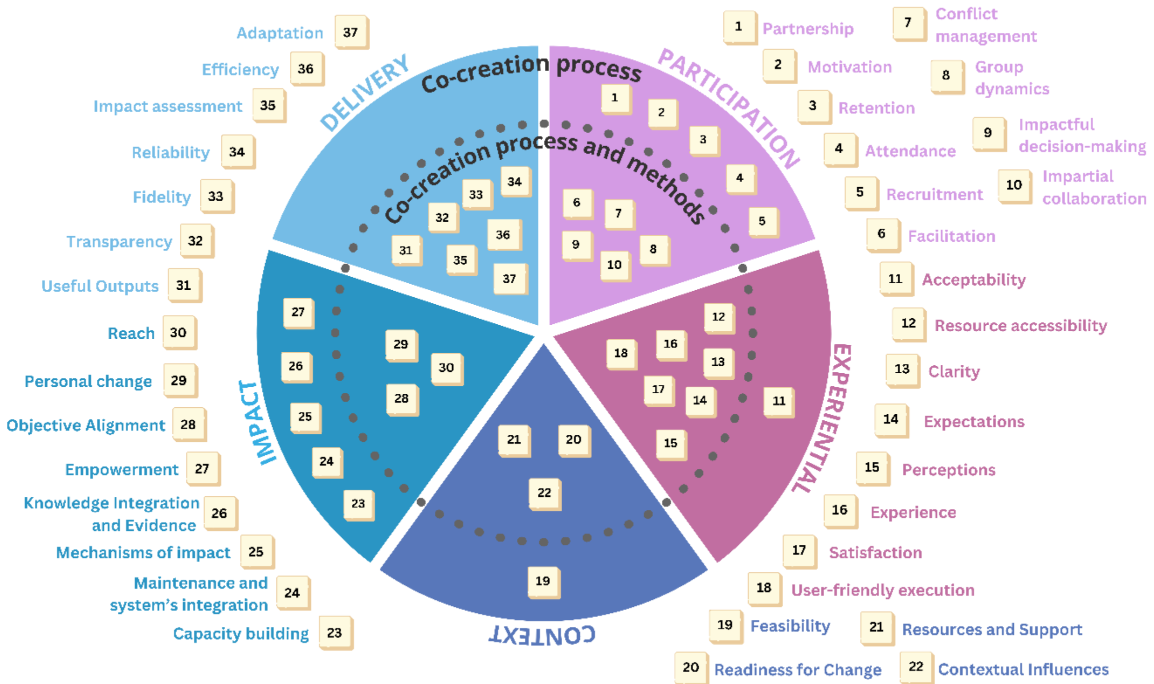

KEY:
Co-creation process = evaluation components that can be used to evaluate the overall co-creation process
Co-creation process and methods = evaluation components that can be used to evaluate the overall co-creation process and the methods used within the co-creation sessions

Figure 4. *Overview of dimensions and components identified through included studies (Figure 2, Longworth & Agnello et al.[49])*

The framework allows researchers and practitioners to select relevant components based on their specific evaluation objectives without implying a hierarchy or requiring the use of all elements. For example, the evaluation might consider co-creators' acceptability and satisfaction alongside the adaptability and feasibility of the process. Capacity building, empowerment, and group dynamics can be assessed together with factors such as clarity, conflict management, and contextual influences. Elements like resource accessibility, transparency, and the usefulness of outputs help assess the fairness and effectiveness of the process, while components such as fidelity, retention, and impact assessment ensure alignment with goals and long-term engagement.

Continuous reflection and iteration

The need for formative evaluation in co-creation has been argued to be crucial to co-creation processes. Van Dijk-de Vries 2019 argues that "to ensure that the end user's perception is continuously captured, researchers should assess stakeholders' needs and involvement in the process throughout the implementation so that, in case of adjustment is needed, implementation mechanisms and ways can be undertaken. If an evaluation is not conducted from an earlier stage, it becomes complex to potentially adapt interventions."[50]

An evaluation is intended to be formative when "data are fed back to the implementation team and/or staff in the target system during the study to adapt and improve the process of implementation during the protocol."[51]

As highlighted by Leask et al., 'iteration' is defined as a cyclical process, which happens at all stages of the co-creation, starting at the very beginning, from your pre-planning stages until your last stage of the process.[3] Understanding the evaluation as iterative breaks the linearity of the process with the scope to ensure participants' needs and beliefs are represented.

Moore and colleagues similarly argue that "as co-creation is an iterative process, evaluation may be embedded throughout the process to ensure the process results in an outcome that is representative of co-creators opinion and suitable, tailored and valid for end-users."[52]

Therefore, **when evaluating the co-creation process** remember to consider evaluating the process by assessing the co-creator's satisfaction and ensuring the developed intervention is representative of the co-creators needs.[3]

Principle 6: Evaluating the co-created intervention

When evaluating the co-creation intervention Leask et al. 2019 suggest you may want to consider evaluating your outcomes by including them in a clinical trial.[3] Several published studies combined the co-creation process with randomised controlled trials. Latomme et al. 2021[53]; Anselma et al. 2019[54]; Emma Cedstrand et al. 2020[55] and Maria Giné Garriga et al. 2019.[56]

When evaluating the co-created intervention, process evaluation has been argued as increasingly essential to capture how interventions are adopted, adapted, and integrated within their intended context. Process evaluation conducted at the implementation stage is well-established and commonly conducted alongside clinical trials. In the last decade, process evaluation has broadened its scope to explore impact mechanisms, as suggested by the United Kingdom Medical Research Council guideline.[57] Frameworks for process evaluation, such as The RE-AIM Framework[58] or Process

Evaluation of Complex Interventions: Medical Research Council Guidance[52] can be sourced from the Longworth et al. 2024 article.[49]

Method Categories for Evaluation:

What type of evaluation data?

In formative evaluation "data are fed back to the implementation team and/or staff in the target system during the study to adapt and improve the process of implementation during the course of the protocol."[51] Data for formative evaluation can include qualitative and quantitative data and the choice of preferred methods for co-creation would depend on which target group you're engaging with, and the co-creation setting.

The following are suggested methods categories for evaluation of the intervention or process:

1. **Assessment:** These methods are mainly used in the Planning Stage, but this category represents one method that can be used for evaluation.
    a. Respondent Validation: This method aims to validate responses from respondents of interviews or surveys — to ensure the interpretation by the facilitator was correct. This can be done using extended focus group discussions where co-creators are presented with interpretations of the data as preliminary findings, and this functioned as a basis for discussion. It was very important to check that what I was inferring [was] what people were meant to say. These were also recorded and transcribed.[3,59]
2. **Interviews:** This category represents different types of interview methods that can be used to evaluate written, verbal, or visual communication material to assess the co-creation process, or the co-created intervention.
    a. Qualitative interviews: qualitative research interviews are either unstructured, semi-structured, lightly structured, or in-depth. Unstructured interviews are suggested in conducting long-term fieldwork and allow respondents to let them express themselves in their ways and pace, with a minimal hold on respondents' responses.[42]
    b. Qualitative content analysis: this method is for systematically evaluating written, verbal, or visual communication material - to keep the systematic nature of the content analysis without quantification. Raw data from transcripts of an audio recording of sessions or interviews.[60]
3. **Reflection:** Reflection has been used as a method for research processes and experiences, in ways that recognise the assumptions, frameworks and patterns of thought and behaviour that

shape thinking and action. This category contains some methods that can be used for evaluating the co-creation process.

a. Co-created River: this is a method used in establishing the co-creation principles and a way for the co-creators to evaluate the co-creation process. Participants are first asked to record on red and yellow sticky notes their challenges (red) and successes (yellow). These were placed in chronological order on a long piece of paper on the workshop space floor, with participants allowed to read and respond to each other's notes.[45]

b. Graffiti wall: this is a method for asking co-creators to respond to particular questions or ideas, as well as providing more flexible space.[45]

4. **Questionnaires / Surveys:** This category represents different survey types you can use to assess your target population and/or your co-creator's experience of the co-creation process or the co-created intervention.

   a. Online survey: this is a quantitative or qualitative surveying method. Various online surveying tools can be used to execute this method.[61]

   b. Process evaluation questionnaire: this method is for the evaluation of individual, collective or management perceptions and actions in implementing any intervention and their influence on the overall result of the intervention.[62]

Evaluating co-creation methods and process

Further research was conducted to explore how to evaluate the methods used during the co-creation process. In a study by Agnello et al. 2024, fifteen criteria were identified for assessing individual co-creation methods. These criteria were used to develop the Observation Guide: Evaluating a Co-Creation Workshop—a practical checklist tool designed to support real-time evaluation of co-creation workshops.[63]

The guide enables an observer to assess each method used in a workshop against relevant evaluation criteria (not all criteria will apply to every method). Using a checklist format, it outlines key elements to monitor, allowing for quick documentation of both facilitator and participant performance in relation to the process and outcomes of each method. It also includes space for brief comments to provide additional context beyond the checklist items.[63] These items have been included and reported in the PROSECO framework, which can be consulted for both an evaluation guidance of the overall co-creation process and methods used throughout[48].

Stage 4: Reporting

Reporting Template

The PRODUCES Framework may be used to report back on your co-created intervention and the co-creation process. In the same way that PRISMA is used as a model to report on systematic reviews, the PRODUCES framework can serve a similar purpose in co-creation.

You can find the PRODUCES+ Reporting Template adapted from Leask and colleagues' paper in Annexe 1. This template can serve as a starting point for reporting on your co-creation process, intervention, and this guideline. Therefore, we encourage you to use this template to report on your experience with using this guideline in your co-creation process, so we can also use your report to improve this guideline and expand its content and structure.

Method for reporting:

One example of a reporting method is *Our Voic*e from Citizen Science used as a citizen science-based community activation and engagement model.[64] However, methods for reporting are currently limited, so at this stage, it is recommended to follow the PRODUCES+ reporting template mentioned above.

Scaling a co-created intervention

Following the four stages of the co-creation process, it is important to consider how the co-created intervention can be scaled. For this guideline, scaling has been defined as the intention to transfer a developed solution to a wider and/or different target population to achieve a greater impact.

How Might Scaling Look?

Scaling might prove particularly challenging in co-creation as the intervention is intended to be designed locally and with individual stakeholders. This "tailored" intervention process might be more difficult to replicate when it provides a highly contextual solution. Scaling and scaling methods are an aspect that has been understudied in co-creation literature, and few resources exist on the topic. Therefore, this will be investigated further, with potential findings included in the next iteration of this guideline. For now, we recommend considering the following three models for scaling proposed by Leask et al.[3]

<u>Three types of scaling models:</u>

The following are the three models that can be used to scale the co-created solution. They are also visualised in Figure 5.

1. Distributed model: "In the distributed model, the design and implementation of each intervention are developed and initiated locally in collaboration with or by local actors specifically for this local group and setting. In this model, local solutions from multiple locations are developed independently and clustered to reach a larger population. Here, the actual co-creation process can be used as an intervention itself to assess whether engaging in the development process has influenced the end-users targeted health behaviour."[3]

2. Generalisable model: "The generalisable model is to develop, in collaboration with a sample of stakeholders and representative end-users of a larger population, a tailored intervention that can be scaled and implemented in a larger group."[3]

3. CASCADE Model: In Leask and colleagues' description of the cascade model, "one local intervention is designed and implemented locally in collaboration with or by local stakeholders and end-users specifically for this local group and settings. This solution is then transported and adapted in collaboration with or by a new group of local stakeholders and end-users for the same purpose, in different settings."[3]

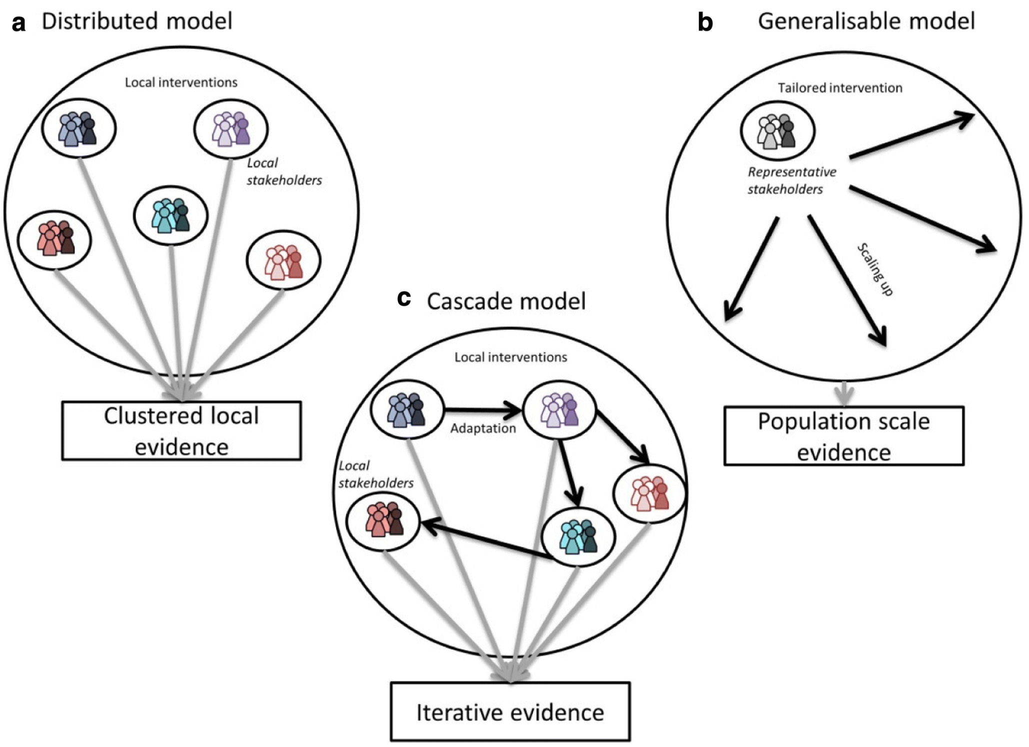

Figure 5. *Models for scaling locally co-created public health interventions using participatory methodologies: a distributed model; b generalisable model; c cascade model (Source = Leask et al. 2019 Figure 4*[3]*)*

Adapting a co-created intervention

While co-creation processes and interventions can be scaled, they may also require adaptation—either to support scaling or to enable transfer and implementation in new settings. Plans for long-term maintenance or scale should be developed through a process that includes adaptation, which may apply to the co-creation process itself or the co-created intervention.[65]

Adapting co-creation processes or public health interventions helps improve their fit with the population of interest, potentially resulting in more relevant and effective outcomes. A recent scoping review by de Boer, Longworth, Delfmann et al. 2025 explored co-adaptation in public health interventions. Although most studies did not provide detailed reporting on the co-adaptation process, some included information on group composition, duration and methods used.[66] The review found that 70 per cent of case studies applied the ADAPT Guidance,[65] while 14 per cent used the Intervention Mapping Adapt framework.[67,68]

Interviews conducted as part of the review highlighted the importance of ensuring contextual fit, integrating prior knowledge and keeping a clear record of adaptations made. These findings emphasise the need for tailored frameworks to support co-adaptation and for improved reporting of co-adaptation processes.

The following frameworks can support you in reflecting on and planning the adaptation of your co-creation process or co-created solution:

- **The ADAPT Guidance:** is used for reporting adaptations and modifications to evidence-based intervention.[65]
- **Intervention Mapping Adapt**: a six-step systematic approach to guide decision-making in each phase of adapting an EBI. The six steps are as follows: assess needs, search for EBIs, assess fit and plan adaptions, make adaptions, plan for implementation and plan for evaluation.[67,68]

This study discovered that co-adaptation was approached differently across the studies included in the rapid review and interview studies, highlighting the diverse nature of the process and the challenges involved when reviewing co-adaptation. Figure 6 visualises various modes of adaptation, i.e., approaches to adapting co-creation processes and/or interventions.

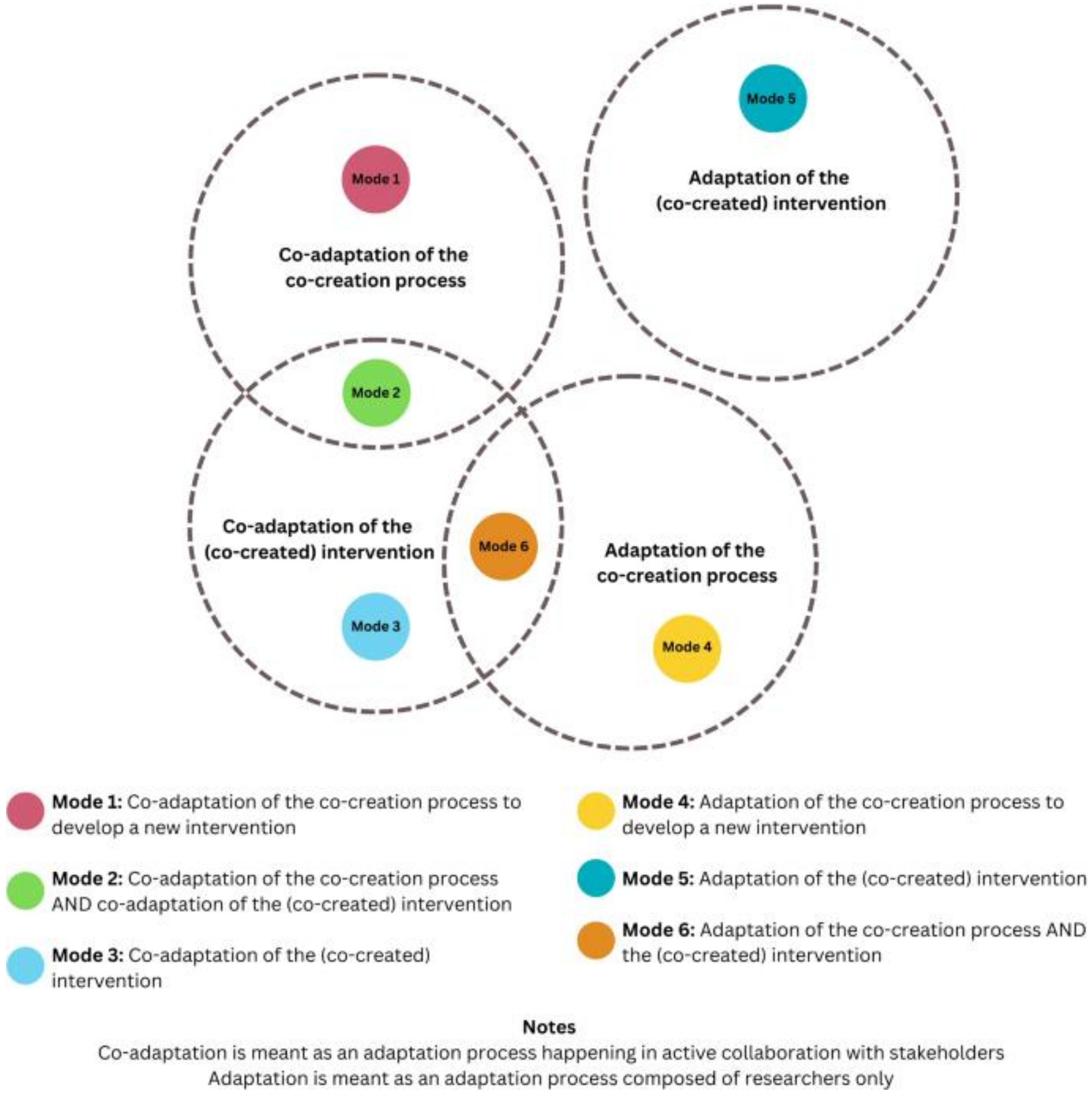

Figure 6. *Modes of Adaptation (Source = de Boer, Longworth, Delfmann et al. 2025, Figure 2 [66])*

7. Conclusion

This document offers a mapping of recent findings, existing resources, frameworks, and methods that we believe are relevant for the planning and conducting of co-creation for public health. This guideline should be considered a prototype to be assessed in the field by the facilitators, researchers, end-users and co-creators.

Annexe 1: PRODUCES+ Planning or Reporting Template

The following reporting template is adapted from Leask et al. 2019 publication, Reporting Checklist — Table 6.[3]

Stage	Checklist Item	Response	Example
**Stage 1: Planning**			
**How was the aim of the study framed?**	Use each element of the PRODUCES framework (PRoblem, Objective, Design, (end-) Users, Co-creators, Evaluation and Scalability)<br><br>Which frameworks did you use?	**Problem**:<br>**Objective**:<br>**Design**:<br>**End-Users**:<br>**Co-Creators**:<br>**Evaluation**:<br>**Scalability**:	Utilising PAAR (Design) to develop (Objective) and test (Evaluation), with academic researchers and older adults (Co-creators), a generalisable (Scalability) intervention to reduce sedentary behaviour (PRoblem) in community-dwelling older adults (end-Users).
**Planning Methods**	Which methods did you use? Why?	**Method 1:**<br>**Method 2:**<br>[*please add in any additional methods*]	Example method: Opportunistic sampling or Asset Assessment.
**Planning Frameworks**	Which frameworks did you use? Why?	**Framework 1:**<br>**Framework 2:**<br>[*please add in any additional frameworks*]	Example frameworks: PRODUCES or PICO framework
**Explain the sampling procedure**	Explain the criteria used for sampling:	**Method 1:**<br>**Method 2:**	Convenience sampling and maximum variation sampling. End-users were 65+ years of

		[*please add in any additional methods*]	age, community-dwelling, able to ambulate independently, able to give informed consent, able to attend a minimum of 5 meetings
	In what settings did sampling occur? What mode of delivery did you use?		End-user co-creators were recruited from the university older adult database
	How many individuals were engaged as co-creators?		Four university researchers and 11 community-dwelling older adults
	Please describe the co-creators (e.g., *demographics/groups/ other characteristics of interest*):		Of the end-user co-creators, 11 participants (5 men), average age = 74 years. Average medications = 5.
**Stage 2: Conducting**			
**How was ownership manifested?**	Please explain the methods used to manifest ownership (e.g., *branding the group, identifying the rights and responsibilities of the group*):	**Method 1:**<br>**Method 2:**<br>[*please add in any additional methods*]	Co-creators branded as GrandStand Research Group. Co-creators provided t-shirts, lab books, bags, and pens with the GrandStand logo. All co-creators told of their right to equal status within the group and their responsibility to contribute their ideas.
**Procedure Components**	What level of participation did you achieve with the co-creators,	**Which level:**<br>**How you determined this:**	Academic researchers and end-users strove to have equal

	according to Arnstein's ladder of citizen participation: *Manipulation, Therapy, Informing, Consultation, Placation, Partnership, Delegated power, or Citizen control* [69]?<br><br>How did you determine this?		participation. All co-creators asked for their input on each discussion point.
	How was the overall aim presented?		The overall aim is highlighted at the beginning of the process and the beginning of each workshop.
	How was the purpose of each meeting presented?		The purpose of each meeting is identified at the beginning of the meeting.
	What were the rules and responsibilities of participation agreed upon?		Individuals are told of their right to equal status within the group and to contribute their ideas.
**Conducting Methods**	Which methods did you use in your conducting stage (*e.g., during your workshops*)?	**Method 1:**<br><br>**Method 2:**<br>**[***please add in any additional methods***]**	Example methods: Concept Mapping or Dialogic Literary Gatherings.
**Procedure Methods**	Which areas did the co-creators require upskilling? How did you upskill them?	**Areas for upskilling:**<br><br>**Upskilling approach:**	End-users were up-skilled regarding behaviour change theory and research methods. Academic researchers were upskilled regarding older adults' reasons and preferences for interrupting SB.

	Was relevant evidence reviewed to support this co-creation process? If yes, how?		Presentations of the context of older adults' SB [93], behavioural assets which can be used to interrupt sedentary periods [94] and behaviour change theories
	If a prototype was developed, please describe the prototype and the prototyping process:	**The Prototype(s):**<br>**Prototyping Process:**	A full intervention prototype was created from several key components which were individually prototyped, tested by co-creators, and then refined.
	Please describe the frequency and duration of meetings and/or workshops throughout the co-creation process:		Meetings occurred every 10–14 days and lasted approximately 2 h.
	Please provide examples of how iteration occurred during your co-creation process:		Prototypes were initially developed, tested externally and after discussions, refined and then tested again.
**Conducting Frameworks**	Which frameworks did you use?	**Framework 1:**<br>**Framework 2:**<br>[*please add in any additional frameworks*]	Example Framework: PRODUCES framework
	Were there any frameworks or models you used that are not mentioned in this guideline? If yes, please list them:		

Stage 3: Evaluating			
**Evaluating Methods**	Which methods did you use?	**Method 1:**<br>**Method 2:**<br>[*please add in any additional methods*]	Example Method: Qualitative Content Analysis
**Evaluating Frameworks**	Which frameworks did you use?	**Framework 1:**<br>**Framework 2:**<br>[*please add in any additional frameworks*]	Example Framework: The PROSECO framework, The APEASE evaluation framework
**Process**	Please explain how co-creator satisfaction and contribution are evaluated (*e.g., reporting on attendance rates, questionnaires, and interviews*).		Retention rates measured (100% retention, 0% dropout)
	How are results reported back to the co-creators and other stakeholders?		
**Outcome**	Please explain how the validity of the outcome and the process were evaluated (*e.g., face validation, and member checking*):		Face validation and member checking occurred throughout, including each developed prototype component and a summary of the information gathered from the previous meeting
	Please explain plans for formal testing of the effectiveness/scalability of the co-created outcome:		Plan to embed the intervention into a multi-centre RCT vs. a top-down, theory-driven intervention and standard care (control group) to assess the

			effectiveness of the intervention.
	Please explain the outcome of the evaluation (if tested):		If you plan for form testing, outcomes which will be measured will include changes in sedentary time, changes in sedentary time fragmentation, participants’ experience of using the intervention and the effect on function (noted as important by the end-user co-creators.
**Any other Comments**	Please input any other details or reflections you think are important to report on your co-creation process:		

References

1. Williams O, Sarre S, Papoulias SC, et al. Lost in the shadows: reflections on the dark side of co-production. doi:10.1186/s12961-020-00558-0

2. Verloigne M, Altenburg T, Cardon G, et al. Making co-creation a trustworthy methodology for closing the implementation gap between knowledge and action in health promotion: the Health CASCADE project. *Perspect Public Health*. 2023;143(4):196-198. doi:10.1177/17579139221136718

3. Leask CF, Sandlund M, Skelton DA, et al. Framework, principles and recommendations for utilising participatory methodologies in the co-creation and evaluation of public health interventions. *Res Involv Engagem*. 2019;5(1):2. doi:10.1186/s40900-018-0136-9

4. van Woezik AFG, Braakman-Jansen LMA, Kulyk O, Siemons L, van Gemert-Pijnen JEWC. Tackling wicked problems in infection prevention and control: a guideline for co-creation with stakeholders. *Antimicrob Resist Infect Control*. 2016;5(1):20. doi:10.1186/s13756-016-0119-2

5. Vargas C, Whelan J, Brimblecombe J, Allender S. Co-creation, co-design, co-production for public health – a perspective on definition and distinctions. *Public Health Res Pract*. 2022;32(2). doi:10.17061/phrp3222211

6. Agnello DM, Loisel QEA, An Q, et al. Establishing a Health CASCADE–Curated Open-Access Database to Consolidate Knowledge About Co-Creation: Novel Artificial Intelligence–Assisted Methodology Based on Systematic Reviews. *J Med Internet Res*. 2023;25(1):e45059. doi:10.2196/45059

7. Messiha K, Chinapaw MJM, Ket HCFF, et al. Systematic Review of Contemporary Theories Used for Co-creation, Co-design and Co-production in Public Health. *J Public Health*. Published online May 5, 2023:fdad046. doi:10.1093/pubmed/fdad046

8. An Q, Sandlund M, Agnello D, et al. A scoping review of co-creation practice in the development of non-pharmacological interventions for people with Chronic Obstructive Pulmonary Disease: A health CASCADE study. *Respir Med*. 2023;211:107193. doi:10.1016/j.rmed.2023.107193

9. Longworth GR, Erikowa-Orighoye O, Anieto EM, et al. Conducting co-creation for public health in low and middle-income countries: a systematic review and key informant perspectives on implementation barriers and facilitators. *Glob Health*. 2024;20(1):9. doi:10.1186/s12992-024-01014-2

10. Agnello DM, Balaskas G, Steiner A, Chastin S. Methods Used in Co-Creation Within the Health CASCADE Co-Creation Database and Gray Literature: Systematic Methods Overview. *Interact J Med Res*. 2024;13(1):e59772. doi:10.2196/59772

11. Chrifou R. *Ethics in Co-Creation for Public Health: A Preliminary, Multi-Source Perspective*. Zenodo; 2022. doi:10.5281/zenodo.7304033

12. Messiha K, Altenburg TM, Schreier M, et al. Enriching the evidence base of co-creation research in public health with methodological principles of critical realism. *Crit Public Health*. 2024;34(1):1-19. doi:10.1080/09581596.2024.2371323

13. Messiha K, Altenburg TM, Giné-Garriga M, Chastin S, Chinapaw MJM. Enriching the Existing Knowledge About Co-creation: Identifying Dimensions of Co-creation Using Explicit Theory in Various Research Fields. *Minerva*. Published online February 17, 2025. doi:10.1007/s11024-024-09559-7

14. Agnello DM, Anand-Kumar V, An Q, et al. Co-creation methods for public health research — characteristics, benefits, and challenges: a Health CASCADE scoping review. *BMC Med Res Methodol*. 2025;25(1):60. doi:10.1186/s12874-025-02514-4

15. Cascade H. *Co-Creation Methods (Infographic)*. Zenodo; 2022. doi:10.5281/zenodo.7414470

16. Agnello DM, An Q, de Boer J, et al. Developing and Validating the Co-Creation Rainbow Framework: Assessing Whether Methods Enact Co-Creation Characteristics in a Mixed-Methods Health CASCADE Study. Published online December 16, 2023. doi:10.5281/zenodo.10391410

17. Longworth GR, Goh K, Agnello DM, et al. A review of implementation and evaluation frameworks for public health interventions to inform co-creation: a Health CASCADE study. *Health Res Policy Syst*. 2024;22(1):39. doi:10.1186/s12961-024-01126-6

18. Smith N, Georgiou M, Jalali MS, Chastin S. Planning, implementing and governing systems-based co-creation: the DISCOVER framework. *Health Res Policy Syst*. 2024;22(1):6. doi:10.1186/s12961-023-01076-5

19. Cascade H. *Co-Creation Decision Making Tool*. Zenodo; 2022. doi:10.5281/zenodo.7414448

20. Kitson AL, Rycroft-Malone J, Harvey G, McCormack B, Seers K, Titchen A. Evaluating the successful implementation of evidence into practice using the PARiHS framework: theoretical and practical challenges. *Implement Sci*. 2008;3(1):1. doi:10.1186/1748-5908-3-1

21. Kitson A, Harvey G, McCormack B. Enabling the implementation of evidence based practice: a conceptual framework. *BMJ Qual Saf*. 1998;7(3):149-158. doi:10.1136/qshc.7.3.149

22. PRECEDE-PROCEED Model - Rural Health Promotion and Disease Prevention Toolkit. Accessed March 21, 2025. https://www.ruralhealthinfo.org/toolkits/health-promotion/2/theories-and-models/precede-proceed

23. Nilsen P. Making sense of implementation theories, models and frameworks. *Implement Sci*. 2015;10(1):53. doi:10.1186/s13012-015-0242-0

24. Atkins L, Francis J, Islam R, et al. A guide to using the Theoretical Domains Framework of behaviour change to investigate implementation problems. *Implement Sci*. 2017;12(1):77. doi:10.1186/s13012-017-0605-9

25. Harvey G, Kitson A. PARIHS revisited: from heuristic to integrated framework for the successful implementation of knowledge into practice. *Implement Sci*. 2016;11(1):33. doi:10.1186/s13012-016-0398-2

26. Moullin JC, Sabater-Hernández D, Fernandez-Llimos F, Benrimoj SI. A systematic review of implementation frameworks of innovations in healthcare and resulting generic implementation framework. *Health Res Policy Syst*. 2015;13(1):16. doi:10.1186/s12961-015-0005-z

27. Michie S, van Stralen MM, West R. The behaviour change wheel: A new method for characterising and designing behaviour change interventions. *Implement Sci*. 2011;6(1):42. doi:10.1186/1748-5908-6-42

28. O'Cathain A, Croot L, Sworn K, et al. Taxonomy of approaches to developing interventions to improve health: a systematic methods overview. *Pilot Feasibility Stud*. 2019;5(1):41. doi:10.1186/s40814-019-0425-6

29. Wilkinson H, Hills D, Penn A, Barbrook-Johnson P. Building a system-based Theory of Change using Participatory Systems Mapping. *Evaluation*. 2021;27(1):80-101. doi:10.1177/1356389020980493

30. Barbrook-Johnson P, Penn AS. *Systems Mapping: How to Build and Use Causal Models of Systems*. Springer International Publishing; 2022. doi:10.1007/978-3-031-01919-7

31. Skarlatidou A, Suskevics M, Göbel C, et al. The Value of Stakeholder Mapping to Enhance Co-Creation in Citizen Science Initiatives. *Citiz Sci Theory Pract*. 2019;4(1). Accessed February 9, 2024. https://oro.open.ac.uk/79358/

32. Etikan I, Musa SA, Alkassim RS. Comparison of Convenience Sampling and Purposive Sampling. *Am J Theor Appl Stat*. 2015;5(1):1-4. doi:10.11648/j.ajtas.20160501.11

33. Deserti A, Real M, Schmittinger F, eds. *Co-Creation for Responsible Research and Innovation: Experimenting with Design Methods and Tools*. Vol 15. Springer International Publishing; 2022. doi:10.1007/978-3-030-78733-2

34. Team Purpose & Culture. HI Toolbox. Accessed April 25, 2024. https://toolbox.hyperisland.com/team-purpose-culture

35. Bruder AM, Crossley KM, Mosler AB, Patterson B, Haberfield M, Donaldson A. Co-creation of a sport-specific anterior cruciate ligament injury risk reduction program for women: A concept mapping approach. *J Sci Med Sport*. 2020;23(4):353-360. doi:10.1016/j.jsams.2019.10.019

36. Trochim WM, McLinden D. Introduction to a special issue on concept mapping. *Eval Program Plann*. 2017;60:166-175. doi:10.1016/j.evalprogplan.2016.10.006

37. UXPin. UX Honeycomb – 7-Factor Design Framework for Great User Experience. Studio by UXPin. August 5, 2024. Accessed March 21, 2025. https://www.uxpin.com/studio/blog/ux-honeycomb-definition-and-use/

38. Mansson L, Wiklund M, Öhberg F, Danielsson K, Sandlund M. Co-Creation with Older Adults to Improve User-Experience of a Smartphone Self-Test Application to Assess Balance Function. *Int J Environ Res Public Health*. 2020;17(11):3768. doi:10.3390/ijerph17113768

39. Ruiz-Eugenio L, Toledo del Cerro A, Gómez-Cuevas S, Villarejo-Carballido B. Qualitative Study on Dialogic Literary Gatherings as Co-creation Intervention and Its Impact on Psychological and Social Well-Being in Women During the COVID-19 Lockdown. *Front Public Health*. 2021;9. doi:10.3389/fpubh.2021.602964

40. Lund A, Holthe T, Halvorsrud L, et al. Involving older adults in technology research and development discussions through dialogue cafés. *Res Involv Engagem*. 2021;7(1):26. doi:10.1186/s40900-021-00274-1

41. Popp J, Carl J, Grüne E, Semrau J, Gelius P, Pfeifer K. Physical activity promotion in German vocational education: does capacity building work? *Health Promot Int*. 2020;35(6):1577-1589. doi:10.1093/heapro/daaa014

42. Jamshed S. Qualitative research method-interviewing and observation. *J Basic Clin Pharm*. 2014;5(4):87-88. doi:10.4103/0976-0105.141942

43. Darnall N, Jolley GJ. Involving the Public: When Are Surveys and Stakeholder Interviews Effective? *Rev Policy Res*. 2004;21(4):581-593. doi:10.1111/j.1541-1338.2004.00095.x

44. Velázquez AL, Galler M, Vidal L, Varela P, Ares G. Co-creation of a healthy dairy product with and for children. *Food Qual Prefer*. 2022;96:104414. doi:10.1016/j.foodqual.2021.104414

45. Bilous R, Hammersley L, Lloyd K. Reflective practice as a research method for co-creating curriculum with international partner organisations. *ResearchGate*. Published online 2018. Accessed March 21, 2025. https://www.researchgate.net/publication/329356487_Reflective_practice_as_a_research_method_for_co-creating_curriculum_with_international_partner_organisations

46. Parsons S, Guldberg ,Karen, Porayska-Pomsta ,Kaśka, and Lee R. Digital stories as a method for evidence-based practice and knowledge co-creation in technology-enhanced learning for children with autism. *Int J Res Method Educ*. 2015;38(3):247-271. doi:10.1080/1743727X.2015.1019852

47. Haffejee F. The use of photovoice to transform health science students into critical thinkers. *BMC Med Educ*. 2021;21(1):237. doi:10.1186/s12909-021-02656-1

48. Longworth GR, Agnello DM, Chastin S, et al. Evaluating the co-creation process in public health interventions: the PROSECO framework. *Public Health*. 2025;245:105783. doi:10.1016/j.puhe.2025.105783

49. Longworth GR, de Boer J, Goh K, et al. Navigating process evaluation in co-creation: a Health CASCADE scoping review of used frameworks and assessed components. *BMJ Glob Health*. 2024;9(7):e014483. doi:10.1136/bmjgh-2023-014483

50. van Dijk-de Vries A, Stevens A, van der Weijden T, Beurskens AJHM. How to support a co-creative research approach in order to foster impact. The development of a Co-creation Impact Compass for healthcare researchers. doi:10.1371/journal.pone.0240543

51. Bauer MS, Damschroder L, Hagedorn H, Smith J, Kilbourne AM. An introduction to implementation science for the non-specialist. *BMC Psychol*. 2015;3(1):32. doi:10.1186/s40359-015-0089-9

52. Moore GF, Audrey S, Barker M, et al. Process evaluation of complex interventions: Medical Research Council guidance. *BMJ*. 2015;350:h1258. doi:10.1136/bmj.h1258

53. Latomme J, Morgan PJ, De Craemer M, Brondeel R, Verloigne M, Cardon G. A Family-Based Lifestyle Intervention Focusing on Fathers and Their Children Using Co-Creation: Study Protocol of the Run Daddy Run Intervention. *Int J Environ Res Public Health*. 2021;18(4):1830. doi:10.3390/ijerph18041830

54. Anselma M, Altenburg T, Chinapaw M. Kids in Action: the protocol of a Youth Participatory Action Research project to promote physical activity and dietary behaviour. *BMJ Open*. 2019;9(3):e025584. doi:10.1136/bmjopen-2018-025584

55. Cedstrand E, Nyberg A, Bodin T, Augustsson H, Johansson G. Study protocol of a co-created primary organizational-level intervention with the aim to improve organizational and social working conditions and decrease stress within the construction industry - a controlled trial. *BMC Public Health*. 2020;20(1):424. doi:10.1186/s12889-020-08542-7

56. Giné-Garriga M, Sandlund M, Dall PM, Chastin SFM, Pérez S, Skelton DA. A Novel Approach to Reduce Sedentary Behaviour in Care Home Residents: The GET READY Study Utilising Service-Learning and Co-Creation. *Int J Environ Res Public Health*. 2019;16(3):418. doi:10.3390/ijerph16030418

57. Skivington K, Matthews L, Simpson SA, et al. A new framework for developing and evaluating complex interventions: update of Medical Research Council guidance. *BMJ*. 2021;374:n2061. doi:10.1136/bmj.n2061

58. Glasgow RE, Vogt TM, Boles SM. Evaluating the public health impact of health promotion interventions: the RE-AIM framework. *Am J Public Health*. 1999;89(9):1322-1327. doi:10.2105/ajph.89.9.1322

59. Holland E. Mentoring communities of practice: what's in it for the mentor? *Int J Mentor Coach Educ*. 2018;7(2):110-126. doi:10.1108/IJMCE-04-2017-0034

60. Selvi AF. Qualitative content analysis. In: *The Routledge Handbook of Research Methods in Applied Linguistics*. Routledge; 2019.

61. Chen T, Drennan J, Andrews L, Hollebeek LD. User experience sharing: Understanding customer initiation of value co-creation in online communities. *Eur J Mark*. 2018;52(5/6):1154-1184. doi:10.1108/EJM-05-2016-0298

62. Abildgaard JS. Frontiers | How to Measure the Intervention Process? An Assessment of Qualitative and Quantitative Approaches to Data Collection in the Process Evaluation of Organizational Interventions. *Front Psychol*. 2016;7. doi:https://doi.org/10.3389/fpsyg.2016.01380

63. Agnello D, Ryom K, Fuglesang Christensen P. Observation Guide: Evaluating a Co-Creation Workshop. Published online November 27, 2024. doi:10.5281/zenodo.14228909

64. Pedersen M, Wood GER, Fernes PK, Goldman Rosas L, Banchoff A, King AC. The "Our Voice" Method: Participatory Action Citizen Science Research to Advance Behavioral Health and Health Equity Outcomes. *Int J Environ Res Public Health*. 2022;19(22):14773. doi:10.3390/ijerph192214773

65. Moore G, Campbell, Copeland L, et al. Adapting interventions to new contexts—the ADAPT guidance | The BMJ. *BMJ*. Published online August 3, 2021. Accessed March 21, 2025. https://www.bmj.com/content/374/bmj.n1679

66. de Boer J, Longworth GR, Delfmann LR, et al. Exploring co-adaptation for public health interventions: insights from a rapid review and interviews. *BMC Public Health*. 2025;25(1):614. doi:10.1186/s12889-025-21544-7

67. Eldredge LKB, Markham CM, Ruiter R, Fernandez M, Kok G, Parcel G. *Planning Health Promotion Programs: An Intervention Mapping Approach, 4th Edition*. Wiley; 2016. Accessed March 21, 2025. https://www.wiley.com/en-us/Planning+Health+Promotion+Programs%3A+An+Intervention+Mapping+Approach%2C+4th+Edition-p-9781119035497

68. Kachingwe ON, Lewis Q, Offiong A, Smith BD, LoVette A, Powell TW. Using the intervention mapping for adaption framework to adapt an evidence-based sexual health intervention for youth affected by trauma. *BMC Public Health*. 2023;23(1):1052. doi:10.1186/s12889-023-15984-2

69. Ladder of Citizen Participation. Organizing Engagement. November 1, 2019. Accessed September 26, 2023. https://organizingengagement.org/models/ladder-of-citizen-participation/
